# Multidrug-resistant *Escherichia coli* causing canine pyometra and urinary tract infections are genetically related but distinct from those causing prostatic abscesses

**DOI:** 10.1038/s41598-024-62028-9

**Published:** 2024-05-24

**Authors:** Parinya Sroithongkham, Naiyaphat Nittayasut, Jitrapa Yindee, Pattaraporn Nimsamer, Sunchai Payungporn, Komkiew Pinpimai, Suppawiwat Ponglowhapan, Pattrarat Chanchaithong

**Affiliations:** 1 Department of Veterinary Microbiology, Faculty of Veterinary Science, Chulalongkorn University, Bangkok, 10330 Thailand; 2 Department of Biochemistry, Center of Excellence in Systems Microbiology, Faculty of Medicine, Chulalongkorn University, Bangkok, 10330 Thailand; 3 Aquatic Resources Research Institute, Chulalongkorn University, Bangkok, 10330 Thailand; 4 Department of Obstetrics, Gynaecology and Reproduction, Faculty of Veterinary Science, Chulalongkorn University, Bangkok, 10330 Thailand; 5 Research Unit in Microbial Food Safety and Antimicrobial Resistance, Chulalongkorn University, Bangkok, 10330 Thailand

**Keywords:** Dogs, *Escherichia coli*, Prostatic abscesses, Pyometra, Multidrug resistance, Virulence, Bacteria, Microbial genetics, Antimicrobial resistance

## Abstract

Despite extensive characterisation of uropathogenic *Escherichia coli* (UPEC) causing urinary tract infections (UTIs), the genetic background of non-urinary extraintestinal pathogenic *E. coli* (ExPEC) in companion animals remains inadequately understood. In this study, we characterised virulence traits of 104 *E. coli* isolated from canine pyometra (n = 61) and prostatic abscesses (PAs) (n = 38), and bloodstream infections (BSIs) in dogs (n = 2), and cats (n = 3). A stronger association with UPEC of pyometra strains in comparison to PA strains was revealed. Notably, 44 isolates exhibited resistance to third-generation cephalosporins and/or fluoroquinolones, 15 were extended-spectrum ß-lactamase-producers. Twelve multidrug-resistant (MDR) strains, isolated from pyometra (n = 4), PAs (n = 5), and BSIs (n = 3), along with 7 previously characterised UPEC strains from dogs and cats, were sequenced. Genomic characteristics revealed that MDR *E. coli* associated with UTIs, pyometra, and BSIs belonged to international high-risk *E. coli* clones, including sequence type (ST) 38, ST131, ST617, ST648, and ST1193. However, PA strains belonged to distinct lineages, including ST12, ST44, ST457, ST744, and ST13037. The coreSNPs, cgMLST, and pan-genome illustrated intra-clonal variations within the same ST from different sources. The high-risk ST131 and ST1193 (phylogroup B2) contained high numbers of ExPEC virulence genes on pathogenicity islands, predominating in pyometra and UTI. Hybrid MDR/virulence IncF multi-replicon plasmids, containing aerobactin genes, were commonly found in non-B2 phylogroups from all sources. These findings offer genomic insights into non-urinary ExPEC, highlighting its potential for invasive infections in pets beyond UTIs, particularly with regards to high-risk global clones.

## Introduction

*Escherichia coli* is a commensal and opportunistic bacterium capable of causing extraintestinal infections with diverse disease patterns in humans, including urinary tract infections (UTIs), neonatal meningitis, and bloodstream infections (BSIs)^1,2^. Uropathogenic *E. coli* (UPEC), a subcategory of the extraintestinal pathogenic *E. coli* (ExPEC) causing UTIs in humans and companion animals, is well-characterised in terms of pathogenicity and antimicrobial resistance (AMR) properties. Uropathogenesis is initiated by compromised urinary function, facilitating ascending translocation to the uroepithelium for colonisation by adhesins such as type 1 fimbriae and P fimbriae. However, these adhesins can also be found in highly pathogenic strains as part of the ExPEC pathotype. Virulence genes (VGs) involved in adhesion (*papA*, *papC*, *sfa*/*focDE*, and *afa*/*draBC*), cytotoxins (*hlyA*, *cnf*, and *vat*), iron acquisition (*fyuA* and *iutA*), and capsule production (*kpsMII*) are abundant in ExPEC from various extraintestinal infections in humans, such as neonatal meningitis and UTIs^3–5^. Presence of the VGs, such as *yfcV* (yfcV fimbrial protein), *vat* (vacuolating toxin), *fyuA* (yersiniabactin receptor), and *iut* (aerobactin), contributes to effective bladder colonisation in mouse model by highly virulent *E. coli* strains, presumably UPEC^6^. The presence of these genes can be utilised as a predictive tool for UPEC classification, with the limitation that it cannot differentiate UPEC and neonatal meningitis *E. coli* (NMEC)^6^. Determination of ExPEC and/or UPEC would be beneficial in identifying highly pathogenic strains, which typically carry numerous VGs and persist opportunistically in the hosts, leading to recurrent infections.

Apart from causing UTsI in dogs and cats, *E. coli* is a common pathogen responsible for life-threatening or challenging-to-treat infections like pyometra, prostatitis, and BSIs in small animal medicine. These diseases are predisposed by hormonal factors that affect tissue proliferation and compromised tissue barriers, rendering them susceptible to bacterial infections.

Phylogroup B2 is one of the most prevalent lineages of endometrial pathogenic *E. coli* (EnPEC) associated with canine pyometra and endometritis in animals, and it carries VGs similar to those found in UPEC strains^7,8^. However, little is known about virulence factors and genotypes of *E. coli* associated with prostatitis and BSIs in pets.

In veterinary practice, fluoroquinolones (FQs) and third-generation cephalosporins (3GCs) serve as broad-spectrum antimicrobials used for the treatment of acute and systemic bacterial infections, including pyometra and BSIs^9^. FQs, with their capability to permeate the blood-prostate barrier, are the preferred antimicrobial drugs for treating canine bacterial prostatitis^10^. However, the effectiveness of antimicrobial treatment is reduced by infections caused by multidrug-resistant (MDR) bacteria, which encompass extended-spectrum β-lactamase (ESBL)- and AmpC β-lactamase-producing *E. coli*. MDR bacteria develop resistance mechanisms through chromosomal mutations and accumulation of antimicrobial resistance genes (ARGs) located on mobile genetic elements (MGEs) such as plasmids, via horizontal gene transfer^11^. Of paramount concern to public health is the global dissemination of *E. coli* sequence type (ST) 131 carrying *bla*_CTX-15_, which is considered a high-risk clone causing extraintestinal infection in humans^11^. The genome of a canine FQ-resistant EnPEC strain belonging to ST131 was sequenced, revealing a multitude of ARGs and carriage of ExPEC VGs^8^. Recently, high-risk *E. coli* clones have been categorised based on their association with AMR determinants and widespread distribution, including ST38, ST167, ST410, ST641, ST648, ST617, and ST1193. Instances of spillover of these high-risk *E. coli* clones causing canine and feline UTIs have been reported^12^. However, the clonal relationships of MDR ExPEC strains associated with reproductive tract infections and BSIs in dogs and cats remain uncertain. Therefore, this study illustrates the virulence genotypes, AMR properties, and genetic background of *E. coli* isolated from pyometra, prostatic abscesses (PAs), and BSIs in dogs and cats and elucidates the clonal relationships of the MDR non-urinary ExPEC strains with previously characterised high-risk *E. coli* clones isolated from UTIs^12^ using whole-genome sequencing (WGS) and in silico analyses.

## Results

### Phylogroups of *E. coli* isolated from pyometra, prostatic abscesses, and bloodstream infections

Out of 104 *E. coli* isolates, 101 isolates were recovered from dogs suffering from canine pyometra (61 isolates), PAs (38 isolates), and BSIs (2 isolates), and the other three isolates were recovered from cats with BSIs.

One-hundred two isolates were identified as phylogroup B2 (65/102), B1 (16/102), A (10/102), F (5/102), D (2/102), C (1/102), E (1/102), and cryptic clade I/II (2/102), while two isolates were unidentifiable phylogroup. Table 1 presents proportions of *E. coli* phylogroups isolated from pyometra, PAs, and BSIs. Phylogroup B2 was predominant in all disease patterns. The proportions of phylogroup B2 isolated from pyometra and BSIs were significantly higher than in PAs, but phylogroup A isolated from PAs was significantly higher than the others. Only *E. coli* isolates from pyometra exhibited variations across all phylogroups.

**Table 1:** Virulence genes associated with extraintestinal infections in *Escherichia coli* classified into phylogroups and sources.

Phylogroups and sources	No. (%)	Virulence genes^#^												
		Adhesins							Toxins					
		*crl*	*fimA*	*sfa*	*papC*	*yfcV*	*iha*	*afa*	*hlyA*	*hlyE*	*cnf*	*tsh*	*sat*	*vat*
All														
Pyometra	61 (100%)	61	52	37	21	51^a^	6	3	21	45^a^	19	4	13	42
PA	38 (100%)	38	33	15	11	19^b^	0	0	10	17^b^	10	2	3	17
BSI	5 (100%)	5	3	1	0	4^a^^,b^	0	0	0	2^a^^,b^	0	1	2	2
Total	104 (100%)	10	88	53	32	74	6	3	31	64	29	7	18	61
A														
Pyometra	2 (3.3%)*	2	1	0	0	0	0	0	0	0	0	0	0	0
PA	8 (21.0%)**	8	6	0	0	2	0	0	0	0	0	0	0	0
Total	10 (9.6%)	10	7	0^x^	0^x^^,y^	2^x^^,y^	0^x^	0^x^	0^x^^,y^	0^x^	0	0^x^^,y^	0^x^	0^x^
B1														
Pyometra	6 (9.8%)	6	5	0	0	1	0	0	0	1	0	1^a^^,b^	0	1
PA	9 (23.7%)	9	8	1	0	0	0	0	0	0	0	0^b^	0	0
BSI	1 (20.0%)	1	0	0	0	0	0	0	0	0	0	1^a^	0	0
Total	16 (15.4%)	16	13	1^x^	0^y^	1^y^	0^x^	0^x^	0^y^	1^x^	0	2^x^^,y^	0^x^	1^x^
B2														
Pyometra	44 (72.1%)*	44	40	33	19	44	5	1	19	42	17	1	12	39
PA	18 (47.4%)**	18	16	14	11	16	0	0	10	16	10	0	3	16
BSI	3 (60.0%) *	3	3	1	0	3	0	0	0	2	0	0	2	2
Total	65 (62.5%)	65	59	48^y^	30^x^	63^z^	5^x^^,y^	1^x^	29^x^	60^y^	27	1^y^	17^x^^,y^	57^y^
C														
Pyometra	1 (1.6%)	1	1	0	0	0	0	0	0	0	0	0	0	0
Total	1 (1.0%)	1	1	0^x^^,y^	0^x^^,y^	0^x^^,y^	0^x^^,y^	0^x^^,y^	0^x^^,y^	0^x^^,y^	0	0^x^^,y^	0^x^^,y^	0^x^^,y^
D														
Pyometra	1 (1.6%)	1	1	0	0	0	0	0	0	0	0	0	0	0
PA	1 (1.6%)	1	1	0	0	0	0	0	0	0	0	1	0	0
Total	2 (1.9%)	2	2	0^x^^,y^	0^x^^,y^	0^x^^,y^	0^x^^,y^	0^x^^,y^	0^x^^,y^	0^x^	0	1^x^	0^x^^,y^	0^x^
E														
Pyometra	1 (1.6%)	1	0	0	0	0	1	1	0	0	0	0	1	0
Total	1 (1.0%)	1	0	0^x^^,y^	0^x^^,y^	0^x^^,y^	1^y^	1^y^	0^x^^,y^	0^x^^,y^	0	0^x^^,y^	1^y^	0^x^^,y^
F														
Pyometra	3 (4.9%)	3	2	2	0	3	0	1	0	0	0	1	0	0
PA	2 (5.3%)	2	2	0	0	1	0	0	0	1	0	1	0	1
Total	5 (4.8%)	5	4	2^x^^,y^	0^x^^,y^	4^x^^,z^	0^x^^,y^	1^x^^,y^	0^x^^,y^	1^x^	0	2^x^	0^x^^,y^	1^x^
Clade I/II														
Pyometra	2 (3.3%)	2	1	1	1	2	0	0	1	1	1	1	0	1
Total	2 (1.9%)	2	1	1^x^^,y^	1^x^^,y^	2^x^^,z^	0^x^^,y^	0^x^^,y^	1^x^^,y^	1^x^^,y^	1	1^x^	0^x^^,y^	1^x^^,y^
Unidentified														
Pyometra	1 (1.6%)*^,^**	1	1	1	1	1	0	0	1	1	1	0	0	1
BSI	1 (20.0%)*	1	0	0	0	1	0	0	0	0	0	0	0	0
Total	2 (1.9%)	2	1	1^x^^,y^	1^x^^,y^	2^x^^,z^	0^x^^,y^	0^x^^,y^	1^x^^,y^	1^x^^,y^	1	0^x^^,y^	0^x^^,y^	1^x^^,y^

Phylogroups and sources	Virulence genes^#^													
	Iron acquisition systems						Protectins							
	*fyuA*	*irp1*	*iucD*	*iutA*	*chuA*	*iroN*	*bssS*	*iss*	*hmsP*	*kpsII*				
All														
Pyometra	52^a^	52^a^	24	24	52^a^	31	60	37	61	46^a^				
PA	23^b^	23^b^	21	21	21^b^	23	38	27	38	15^b^				
BSI	3^a^^,b^	3^a^^,b^	4	4	4^a^^,b^	2	5	2	5	4^a^^,b^				
Total	78	78	49	49	77	56	103	66	104	65				
A														
Pyometra	0	0	0	0	0	0	2	0	2	0				
PA	2	2	5	5	2	2	8	4	8	1				
Total	2^x^	2^x^	5	5	2^x^^,y^	2	10^x^^,y^	4	10	1^x^^,y^				
B1														
Pyometra	4	4	3	3	1	6	6	6	6	0				
PA	2	2	7	7	0	6	9	8	9	0				
BSI	1	1	1	1	0	1	1	1	1	0				
Total	7^x^	7^x^	11	11	1^y^	13	16^x^^,y^	15	16	0^y^				
B2														
Pyometra	44^a^	44^a^	16	16	44	22	44	24	44	39				
PA	17^a^^,b^	17^a^^,b^	6	6	16	13	18	12	18	12				
BSI	2^b^	2^b^	2	2	3	0	3	0	3	3				
Total	63^y^	63^y^	24	24	63^z^	35	65^x^^,y^	36	65	54^z^				
C														
Pyometra	0	0	0	0	0	0	1	1	1	0				
Total	0^x^	0^x^	0	0	0^x^^,y^	0	1^x^^,y^	1	1	0^x^^,y,z^				
D														
Pyometra	0	0	0	0	1	0	1	1	1	0				
PA	1	1	1	1	1	1	1	1	1	1				
Total	1^x^^,y^	1^x^^,y^	1	1	2^x^^,z^	1	2^x^^,y^	2	2	1^x^^,y,z^				
E														
Pyometra	1	1	1	1	1	0	1	0	1	1				
Total	1^x^^,y^	1^x^^,y^	1	1	1^x^^,y,z^	0	1^x^^,y^	0	1	1^x^^,z^				
F														
Pyometra	1	1	3	3	3	0	2	2	3	3				
PA	1	1	2	2	2	1	2	2	2	1				
Total	2^x^	2^x^	5	5	5^x^^,z^	1	4^x^	4	5	4^x^^,z^				
Clade I/II														
Pyometra	1	1	1	1	1	2	2	2	2	2				
Total	1^x^^,y^	1^x^^,y^	1	1	1^x^^,y,z^	2	2^x^^,y^	2	2	2^x^^,z^				
Unidentified														
Pyometra	1	1	0	0	1	1	1	1	1	1				
BSI	0	0	1	1	1	1	1	1	1	1				
Total	1^x^^,y^	1^x^^,y^	1	1	2^x^^,z^	2	2^x^^,y^	2	2	2^x^^,z^				

^*#*^Virulence genes include *crl*, curli fimbriae; *fimA*, type 1 fimbriae; *sfa*, S fimbriae; *papC*, pyelonephritis-associated pili; *yfcV*, yfcV fimbrial protein; *iha*, iron-regulated gene homologue; *afa*, afimbrial adhesin; *hlyA*, hemolysin A; *hlyE*, hemolysin E; *cnf*, cytotoxin necrotising factor; *tsh*, temperature-sensitive hemagglutinin; *sat*, secreted autotransporter toxin; *vat*, vacuolating autotransporter toxin; *fyuA* and *irp1*, yersiniabactin; *iucD* and *iutA*, aerobactin; *chuA*, heme uptake; *iroN*, salmochelin; *bssS*, biofilm regulator bssS; *iss*, increase serum survival; *hmsP*, putative phosphodiesterase; and *kpsII*, group II capsule antigen.

*^,^** Significantly different frequency of *E. coli* isolates among different diseases within phylogroup (*p* < 0.05).

^a,b^Significantly different frequency of virulence gene carriage among *E. coli* isolated from each source (*p* < 0.05).

^x,y,z^Significantly different frequency of virulence gene carriage within phylogroup (*p* < 0.05).

### Virulence gene carriage

A total of 104 *E. coli* isolates recovered from pyometra, PAs, and BSIs were screened for VG carriage. The isolates that carried ≥ 2 out of 5 VGs (*papC*, *sfa*/*foc*, *afa*, *iutA*, and *kpsII*) were categorised as ExPEC, whereas those carried ≥ 3 out of 4 VGs (*yfcV*, *vat*, *fyuA*, and *chuA*) were classified as UPEC^4,6^. Table 2 presents the proportions of *E. coli* isolated from pyometra, PAs, and BSIs, classified into UPEC and ExPEC pathotypes. A significantly higher proportion of pyometra strains was categorised as UPEC compared to PA. However, proportions of *E. coli* classified as ExPEC were not significantly different. A range of 3–22 VGs were detected in the *E. coli* isolates. VGs associated with UPEC (*yfcV*, *fyuA*, *chuA*), as well as *irp1*, *hlyE*, and *kpsII*, were significantly more prevalent in isolates from pyometra than in PAs. ExPEC VGs, including *papC*, *iha*, *afa*, *hlyA*, and *cnf*, were common in isolates from pyometra and PAs but were not detected in BSI isolates. VGs were associated with the phylogroups in which high numbers of VGs, including *sfa*, *papC*, *yfcV*, *tsh*, *hlyA*, *hlyE*, *vat*, *fyuA*, *irp1*, *chuA*, and *kpsII*, were significantly presented in phylogroup B2.

**Table 2:** *Escherichia coli* (n = 104) isolated from canine (n = 101) and feline (n = 3) extraintestinal infections classified into extraintestinal pathogenic *E. coli* (ExPEC) and uropathogenic *E. coli* (UPEC) genotypes.

Source	No. (%)		
	ExPEC	UPEC	ExPEC and/or UPEC
Pyometra (n = 61)	44 (72.1%)	49 (80.3%)^a^	52 (85.2%)^a^
Prostatic abscess (n = 38)	19 (50.0%)	17 (44.7%)^b^	21 (55.3%)^b^
Bloodstream infection (n = 5)	2 (40%)	3 (60.0%)^a, b^	3 (60%)^a, b^
Total (n = 104)	65 (62.5%)	69 (66.3%)	76 (73.1%)

^a,b^Significantly different proportion of ExPEC and UPEC genotypes among different diseases (*p* < 0.05).

### Antimicrobial resistance and genetic characteristics of 3GC- and/or FQ-resistant *E. coli*

Out of 104 *E. coli* isolates, 44 exhibited FQ resistance (FQ^R^) and 3GC resistance (3GC^R^) (13/44), 3GC^R^ only (5/44), and FQ^R^ only (26/44). These were classified into ESBL (15/44) and AmpC (7/44) phenotypes, as shown in Supplementary Table S1. Regarding ESBL and AmpC production, 15 isolates carried *bla*_CTX-M_, including *bla*_CTX-M-15_ (6/15), *bla*_CTX-M-27_ (4/15), *bla*_CTX-M-55_ (3/15), and *bla*_CTX-M-14_ (2/15). Additionally, seven AmpC-producing isolates carried CIT (5/7), FOX (2/7), and ACC (1/7). The proportions of FQ^R^ and/or 3GC^R^ were high in BSI (5/5) and PA (20/38) isolates, but only 19 out of 61 isolates from pyometra exhibited FQ^R^ and/or 3GC^R^ phenotypes. Overall, 39 out of 44 isolates were MDR, expressing resistance to one drug in three or more antimicrobial groups and carrying multiple ARGs. Frequencies of AMR and ARG carriage, as well as AMR patterns, are provided in Supplementary Figs. S1 and S2, respectively.

Phylogroups of the 44 *E. coli* isolates were identified as follows: B2 (20/44), B1 (10/44), A (9/44), E (1/44), F (3/44), and an unidentified phylogroup (1/44). DNA fingerprint analysis by repetitive extragenic palindromic PCR (REP-PCR) revealed three major clades associated with the phylogroups: clade I-B1 (8/44), clade III-A (6/44), and clade VI-B2 (10/44). The DNA fingerprint pattern of the other isolates was distinct in minor clades (Supplementary Fig. S2). An identical DNA fingerprint pattern was observed for two *E. coli* phylogroup B2 strains, one from pyometra (CUVET21-PYO103) and another from BSI (CUVET21-H2), in the same dog, which was presented in clade VIII.

### Genome characteristics of *E. coli* isolates subjected for WGS analysis

The genome of 12 *E. coli* strains, isolated from pyometra (4 isolates), PAs (5 isolates), and BSIs (3 isolates), as indicated in Supplementary Fig. S2, were sequenced and compared to the genome of seven *E. coli* strains from UTIs. Chromosome sizes ranged from 4,836,704 to 5,583,986 bp and had a G + C content from 50.42 to 50.86%. Chromosome size, the number and size of plasmids, genetic features, as well as the locations of ARGs, for the 19 *E. coli* strains were provided in Supplementary Table S2. All strains contained at least one plasmid, except for strain CUVET21-1783 in ST648 from BSI. Sixteen strains carried MDR plasmids, which contained multiple ARGs.

### Clonal relationship of ExPEC strains from different disease patterns

**Figure 1:**
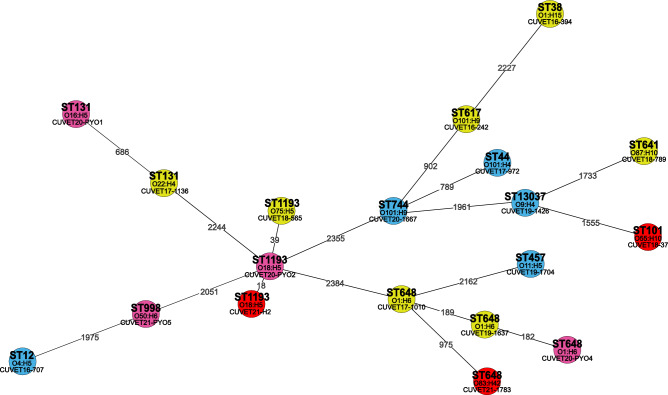
Minimum spanning tree based on core genome multilocus sequence typing (cgMLST) of 19 *Escherichia coli* strains*.* The isolates from pyometra (4 isolates, pink circles), prostatic abscesses (5 isolates, blue circles), bloodstream infections (3 isolates, red circles) and urinary tract infections (7 isolates, yellow circles) in dogs and cats are indicated in individual circles, presenting *E. coli* strains, sequence types (ST), and O:H antigens. Black lines and numbers indicate genetic relatedness and allele differences, respectively.

Three pyometra strains and two BSI strains shared common genetic background of high-risk *E. coli*, including ST131-B2 (ST-phylogroup), ST1193-B2, and ST648-F, with intra-clonal variation. The core genome multilocus sequence typing (cgMLST) analysis revealed only 18 and 39 different loci between the ST1193-B2 strains from BSI and UTI, respectively, compared with the pyometra strain. ST648-F strains from pyometra and UTI were more closely related, as evidenced by a difference of only 182–189 loci, while these strains were distant from the ST648-F strain from BSI with more than 975 loci difference. Up to 686 different loci were observed between ST131-B2 strains from pyometra and UTI. Figure 1 illustrates the minimum spanning tree based on cgMLST of the 19 *E. coli* isolates, representing clonal relationships among and within the high-risk clones.

**Figure 2:**
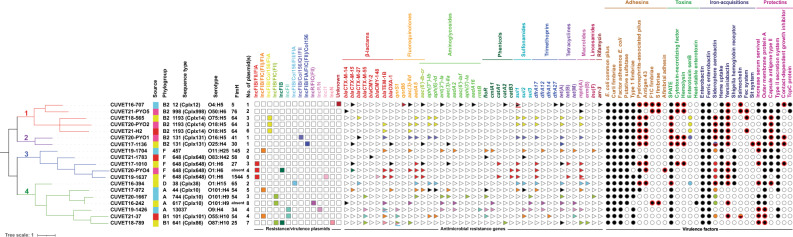
Phylogenetic tree based on core genome single-nucleotide polymorphisms (coreSNPs) of 19 *Escherichia coli* strains. Colour strips indicate sources as follows: pink, pyometra (4 isolates); blue, prostatic abscesses (5 isolates); red, bloodstream infections (3 isolates); and yellow, urinary tract infections (7 isolates) in dogs and cats. Squares and triangles indicate the presence of plasmids and antimicrobial resistance genes (ARGs), respectively. Circles indicate the presence of at least one of the genes responsible for each virulence factor. Black circles and circles enclosed with red borders indicate presence of the genes on chromosome and pathogenicity islands, respectively. The same colour indicates co-localisation on the same plasmid. Genes presenting on both chromosome and plasmid were indicated by the colours and black in the same icon. Icons underlined with blue and red indicate the presence of two and three copies of the gene, respectively. ARGs comprise β-lactamases encoding genes, *bla*_TEM-1_, *bla*_OXA-1_; extended-spectrum β-lactamase encoding gene, *bla*_CTX-M-14_, *bla*_CTX-M-15_, *bla*_CTX-M-27_, and *bla*_CTX-M-55;_ AmpC β-lactamase encoding genes, *bla*_CMY-2_ and *bla*_CMY-148;_ plasmid-mediated quinolone resistance (PMQR) genes, *aac(6′)-Ib-cr*, *qnrS1*, and *qnrB6*; aminoglycoside-modifying enzyme-encoding genes, *aac(3)-IIa*, *aac(3)-IId*, *aac(6′)-Iaf*, *aadA2*, *aadA5*, *aadA16*, *ant(3′′)-Ia*, *aph(3′)-Ia*, *aph(3′′)-Ib*, *aph(6)-Id*, and *rmtB*; florfenicol/chloramphenicol efflux protein genes, *floR* and *cmlA*; chloramphenicol acetyltransferase gene, *catA*; alternative dihydropterate synthase genes, *sul1*, *sul2*, and *sul3*; alternative dihydrofolate reductase genes, *dfrA12*, *dfrA14*, *dfr17*, and *dfrA27*; tetracycline efflux protein genes, *tet*(A), *tet*(B), and *tet*(M); macrolide phosphotransferase gene, *mph*(A); macrolide-resistance ribosomal RNA methyltransferase gene, *erm*(B); lincosamide nucleotidyltransferase gene, *lnu*(F); and rifampin ADP-ribosyltransferase gene, *arr*-3.

The single-nucleotide polymorphisms (SNP) -based phylogenetic tree, based on 2,438 core genes, clustered the *E. coli* strains into four clades (Fig. 2). Phylogroup B2 strains were specific to the closely related clade 1 and 2, while phylogroup F was specific to clade 3. Phylogroup A, B1 and D were separately grouped in clade 4. In addition to 2438 core genes, accessory genes, comprising 8950 cloud genes and 5000 shell genes, were found among this *E. coli* population. Based on the presence and absence of the accessory genes, three clusters specifically grouped the phylogroup B2, F and a group of phylogroup A, B1 and D. Additionally, a strong correlation was observed between the accessory genome and the coreSNP analysis (see Supplementary Fig. S3 online).

### Pathogenicity islands (PAIs) and plasmids

**Figure 3:**
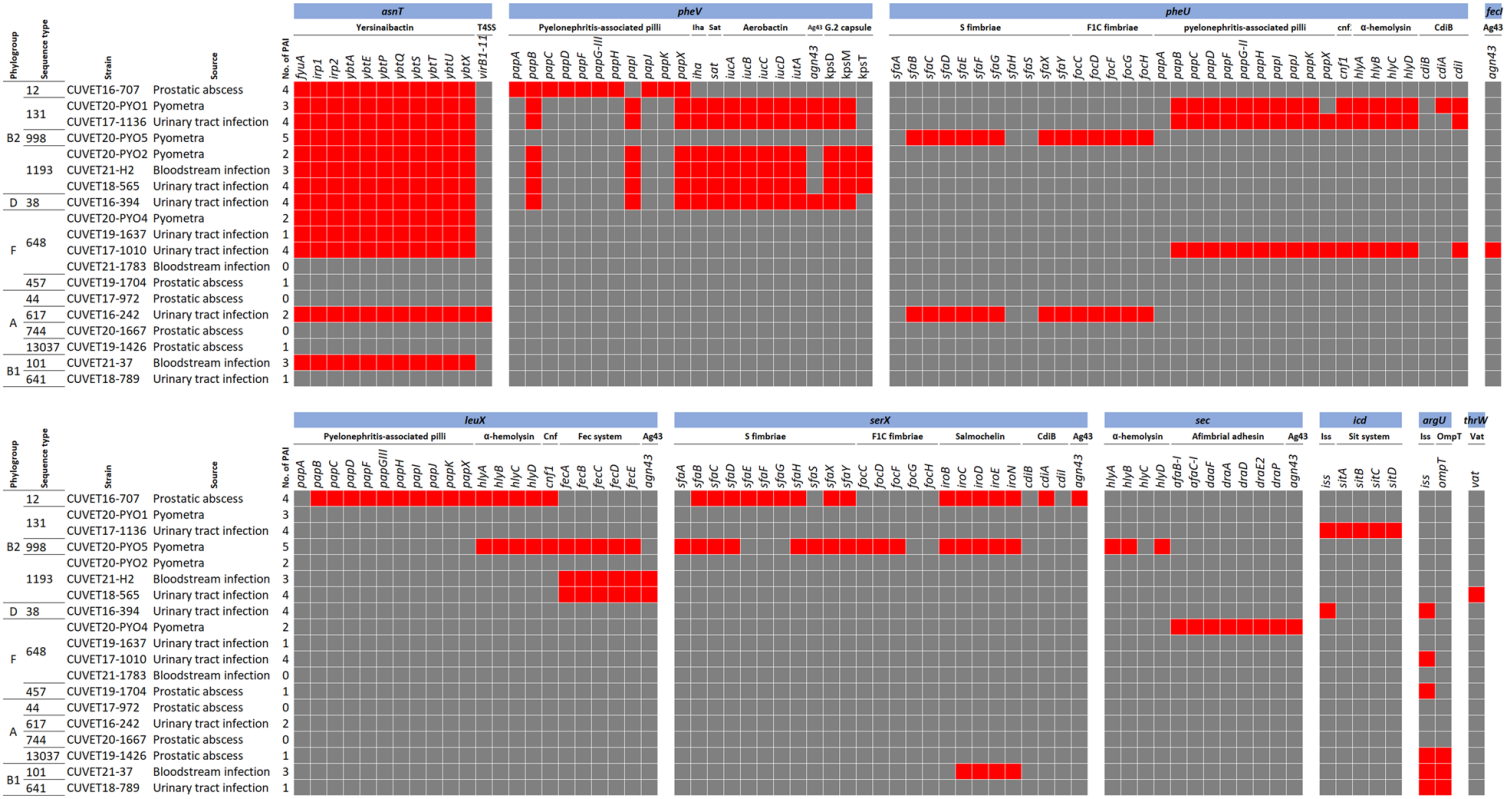
Pathogenicity islands (PAIs) identified in 19 *Escherichia coli* strains*.* PAIs containing virulence genes inserted at the insertion sites of 19 *Escherichia coli* strains isolated from canine pyometra (4 isolates) and prostatic abscesses (5 isolates), and bloodstream infections (3 isolates) and urinary tract infections (7 isolates) in dogs and cats, classified into phylogroups and sequence types (ST). Red squares indicate the presence of virulence genes in each PAI.

PAI(s) were identified on the chromosome of 16 *E. coli* isolates (Fig. 3), and they were absent in three isolates, including ST44-A and ST744-A from PAs, and ST648-F from BSI. The phylogroup B2 isolates contained up to five PAIs that carried several VGs (Fig. 3 and Supplementary Table S3). PAIs that co-harbored *iss* and *ompT* were specifically found in phylogroup A and B1. Common yersiniabactin-associated PAIs, classified as high PAIs (HPI), were present across multiple phylogroups, except for phylogroup A. However, a variant of the yersiniabactin-associated PAIs containing the type IV secretion system (T4SS) was detected in CUVET16-242 of phylogroup A from UTI. Moreover, a PAI containing *sfa/foc* genes was unusually inserted following *phe*U, also found in strain CUVET20-PYO5 (phylogroup B2), which carried the most numerous VGs. The aerobactin-encoding gene cluster was abundantly found on multi-replicon plasmids that carried ARGs, considered hybrid plasmids. The numbers and replicon types of resistance and virulence plasmids are illustrated in Fig. 2. Details of total plasmids and ARG and VG localisation are provided in Supplementary Table S2.

### Virulome and resistome

Among the 19 sequenced *E. coli* isolates, 139 VGs encoding 27 virulence factors, and 41 ARGs mediating resistance to 10 antimicrobial classes were identified (Fig. 2 and Supplementary Fig. S4). Overall, high numbers of VGs were observed in phylogroup B2 (78–97 gene, with an average of 85.3 genes), followed by phylogroup F (63–95 genes, with an average of 76.4 genes). PAI-encoded ExPEC toxin and capsular genes, including *cnf1*, *hlyA*, and *kps*, were specifically present in phylogroup B2 and F isolates. In contrast, VG carriage was fewer in the strains in phylogroup A (35–60 genes, with an average of 51.2 genes) and phylogroup B1 (38–56 VGs with an average of 47 genes). Strain CUVET21-PYO5, belonging to ST998-B2, carried 97 VGs encoding 22 virulence factors but harbored only two ARGs, including *bla*_CTX-M-14_ and *mph*(A) on the chromosome. Strain CUVET19-1426, belonging to ST13037-A isolated from PA, had the lowest VG carriage, containing only 35 genes, but carried the highest number of ARGs on plasmids.

Regarding ARG carriage, phylogroup A isolates carried the highest number (11–17 genes with an average of 13.2 genes), while phylogroup B2 isolates had the lowest number (2–16 genes with an average 8.3 genes). Localisation of ARGs on the chromosome and plasmids in each isolate is indicated in Fig. 2. The majority of ARGs were located on plasmids in all plasmid-carrying isolates, except for ST12-B2. Larger than 120 kb hybrid virulence/MDR multi-replicon plasmids, carrying aerobactin genes, were more common in the phylogroup A and F isolates (Fig. 2 and Supplementary Table S2). Among the 14 *bla*_CTX-M_-positive isolates, six strains harbored *bla*_CTX-M_ on the chromosome, four of which were phylogroup B2. The remaining eight *bla*_CTX-M_-positive isolates contained the gene on IncF plasmids, except for one strain carrying on an IncR/N plasmid. Only within ST648, chromosomal *bla*_CMY-2_ and plasmidic *bla*_CMY-148_ were detected. Among the 19 *E. coli* strains, mutation at *gyrA*:pS83L was detected in 18 strains, but 17 expressed FQ^R^. Three mutations of quinolone resistance-determining regions (QRDR), including *gyrA*:pS83L, *gyrA*:pD87N, and *parC*:pS80I, were observed in 15 FQ^R^ strains. Of these, six strains harbored plasmid-mediated quinolone resistance (PMQR) genes, distributed on the chromosome and plasmids. Despite the absence of mutation of QRDR, one strain in phylogroup B2 contained two PMQR genes mediating FQ^R^, including *aac(6')-Ib-cr* on chromosome and *qnrB6* on a plasmid.

## Discussion

In addition to UTIs, *E. coli* phylogroup B2 was associated with pyometra, PAs, and BSIs and contained the most abundant VGs. The majority of *E. coli* isolated from pyometra, PAs, and BSIs in this study belonged to phylogroup B2, as those isolated from extraintestinal infections in pets and humans in previous studies^8,13–16^. However, the characteristics of BSI isolates should be interpreted with consideration regarding the small number of isolates, which might lead to bias in statistical analysis. The virulence genotyping revealed that 72.1% and 80.3% of the *E. coli* isolated from pyometra contained VGs of ExPEC and UPEC pathotypes^5,6^, respectively, which were likely associated with the phylogroup rather than the disease patterns. Due to the lower numbers of phylogroup B2 in PA strains, ExPEC and UPEC genotypes were infrequently found in *E. coli* isolates from PAs. Non-B2 phylogroups isolated at lower frequencies in all diseases and contained less complete VGs required for the steps of ExPEC pathogenesis. In other extraintestinal infections such as UTIs, non-B2 *E. coli* phylogroups contained fewer VGs; however, genes encoding adhesins and siderophores were detected in most of the isolates^4^. The binding of type 1 fimbriae encoded by *fimA* to uroepithelium and uteroepithelium is considered a critical step in adhesion during uropathogenesis and uteropathogenesis, respectively^17,18^. The presence of specific ExPEC adhesin genes, such as *sfa* encoding S-fimbriae, can support successful extraintestinal colonisation. S-fimbriae specifically bind α-sialyl-2-3-β-galactose (NeuAc-α 2,3-Gal) on the animal cell surface and can enhance adhesion of UPEC^17^. The expression of *fyuA* and *irp1* (yersiniabactin) in alkaline conditions, like urinary bladder and uterus, assists in iron acquisition for extraintestinal survival^6,14,19,20^. Likewise, hemolysin A and capsular genes are more specific to phylogroup B2 in UPEC and NMEC strains^13^. Therefore, the extraintestinal infections frequently caused by *E. coli* phylogroup B2 could be enhanced by all VGs corresponding to adhesion, toxin production, iron acquisition, and immune evasion. However, adhesins and iron acquisition systems were likely required in the extraintestinal pathogenesis of any *E. coli* phylogroups.

According to PAI and virulome analyses, PAIs served as the key genetic determinants for the virulence of the ExPEC isolates. High numbers of VGs, such as capsular, hemolysin A, and pyelonephritis-associated pili genes, were consistently detected on the PAIs found in phylogroup B2 across various disease patterns. Essentially, HPI containing yersiniabactin gene cluster, were non-specifically widespread in all phylogroups, supporting the fundamental requirement of an iron-acquiring system to survive outside the intestine^21^. Evidence supports the notion that pathogenic attenuation of *E. coli* strains containing the HPI can be caused by *irp2* inactivation^22^. In phylogroup B2, the *phe*V-PAI containing iron-regulated gene homologue adhesin (*iha*), secreted autotransporter toxin (*sat*), aerobactin (*iuc* and *iut*), and capsular (*kps*) genes, were conserved in isolates from any infections. Additionally, the *phe*U-PAI containing the *pap* gene cluster, hemolysin A (*hlyA*), cytotoxin necrotising factor (*cnf*), and contact-dependent growth inhibitor (*cdi*), were more specific to ST131-B2. These PAIs bearing VGs associated with uropathogenesis are detected in phylogroup F and D from urinary sources^23^, but not in these phylogroups isolated from PAs, and BSIs in our study. Sharing of ExPEC VGs among the *E. coli* clones could be described by the presence of S fimbriae-associated PAI (*phe*U-PAI) in ST998-B2 and ST617-A. Additionally, the PAI-encoded aerobactin genes (*iutA* and *iuc* gene cluster) in B2 strains were carried on plasmids in non-B2 strains. The acquisition of MGEs encoding ExPEC VGs as part of the accessory genome, such as PAI-encoded *sfa/foc* and plasmid-encoded *iut*/*iuc*, could be considered an evolutionary process leading to ExPEC, as assessed by the ExPEC criteria^6^.

The genomic background of MDR ExPEC from pyometra and BSIs using multilocus sequence typing (MLST) analysis showed a clonal relationship to high-risk ExPEC strains causing UTIs, including ST131-B2, ST1193-B2, and ST648-F, which are important for global monitoring, but PA isolates appeared to be distinct. Canine uterosepsis caused by *E. coli* ST1193-B2 was presented in a dog concurrently suffering from pyometra and BSI. Due to the identical genetic characteristics of strains from the uterus and blood, only the strain isolated from blood (CUVET21-H2) was subjected to WGS, revealing significant VG carriage such as the capsule production gene *kpsII* for immune evasion. *E. coli* ST1193 contains VGs similar to those found in ST131 but commonly carries the *senB* gene on IncF plasmids and is associated with infections in humans in the community rather than nosocomial infections^24^.

Intra-clonal variation was observed in the high-risk STs by coreSNP analysis, cgMLST, variations in O and H antigens, as well as differences in the type and location of accessory genes associated with virulence and resistance. *E. coli* ST131-B2 strains from both pyometra and UTI exhibited the greatest genetic diversity within the same ST. The chromosomally encoded *bla*_CTX-M-15_ *E. coli* ST131 strain associated with UTI belonged to clade C2/H30Rx, which is related to the predominant bacteremic *E. coli* ST131 SEA-C2 clone in Southeast Asia and disseminated worldwide^25^. Furthermore, heterogeneity was observed among ST1193 strains in terms of 3GC^R^ development, illustrating acquisition of *bla*_CTX-M_, which was found in one strain from UTI. The emergence of MDR *E. coli* ST1193 has been increasingly reported, representing a successful global high-risk clone following the footsteps of *E. coli* ST131^26^. In Thailand, ST131, ST648, and ST1193 are the most common STs associated with high-risk ESBL-producing *E. coli* carried by hospitalised patients^27^. High-risk MDR *E. coli* clones dominate the population of 3GC^R^ and FQ^R^ *E. coli*. Selecting 3GC^R^ and FQ^R^ isolates in AMR monitoring of ExPEC in animals could support the findings of high-risk clones of global concern.

Although a low number of 3GC^R^ was observed, the carriage of *bla*_CTX-M_ was found to be common in 3GC^R^ ExPEC associated with high-risk *E. coli* clones from UTIs, and pyometra, as well as in non-high-risk clones from PAs. The detected *bla*_CTX-M_ variants in this study, including *bla*_CTX-M-15,_ *bla*_CTX-M-14_, *bla*_CTX-M-27_, and *bla*_CTX-M-55_, are the most common variants in *E. coli* across humans, livestock, and the environment^28^, suggesting the wide dissemination within the *E. coli* population. The predominant *bla*_CTX-M_ variants were the same as those found in canine and feline UPEC in Thailand^12^. The presence of *bla*_CTX-M_ genes on plasmids is more common; however, chromosomal integration of *bla*_CTX-M_ and AmpC, mediated by IS*Ecp*1, supported the stabilisation of the gene in the genome. This could facilitate clonal dissemination, as found in ST131^29,30^. Mutations in the QRDR region in *gyrA* and/or *parC* are the major mechanism of FQ^R^ in bacteria and a primary factor for the successful clonal spread of ExPEC ST131 and ST1193, contributing to their emergence worldwide^26^. PMQR genes are an alternative mechanism in enterobacteria, developed through the acquisition of MGEs. In this study, most of the ARGs were carried by multi-replicon IncF plasmids, which plays a crucial role in bacterial evolution and primarily influences resistance evolution in these ExPEC strains^31^. The multiple recombination of the IncF plasmids, generating large multi-replicon plasmids containing numerous ARGs, VGs, and transfer modules, results in greater stability within bacterial hosts and more efficient transfer^32,33^. The evolution from IncF plasmid carriage and plasmid recombination might potentially promote *E. coli* to persist outside the gastrointestinal tract through iron acquisition in infections and enhance resistance to antimicrobials during the treatment.

Pan-genome analysis demonstrates the concurrent evolution of core genes by SNPs and accessory genes by gene acquisition in each lineage, indicating co-evolution in both core and accessory genomes. There is a high correlation between the core genome and accessory genome of *E. coli* ST117, reflecting diverse evolution in this clone by acquisition of PAIs and ARGs^34^. Overall, both core and accessory genome of the ExPEC strains are associated with evolution within the lineages and AMR. However, limitations of the study were addressed by the low numbers of WGS-sequenced isolates that were selected based on the highest numbers of virulence and resistance genes among the isolates from different disease patterns, as well as the far fewer isolates from BSIs.

This study highlighted the virulence traits of ExPEC causing canine pyometra and PAs, and BSIs in dogs and cats, most of them belong to phylogroup B2 containing numerous ExPEC VGs similar to those found in UPEC. However, non-B2 phylogroups may at least require adhesion and an iron-acquiring system. The 3GC^R^ and FQ^R^ *E. coli* containing high numbers of ARGs and VGs from pyometra and BSIs are related to three high-risk *E. coli* lineages: B2-ST131, B2-ST1193, and F-ST648. Genetic features of *E. coli* from PAs are distinct from those of other extraintestinal infections. Virulence in the lineages is presented by PAIs in the chromosome, and IncF plasmids, especially multi-replicon plasmids, also play a crucial role in MDR evolution. Thus, pets act as a reservoir of high-risk *E. coli*, posing a risk of transmission to humans in the community. Effective diagnostic antimicrobial susceptibility testing to improve antimicrobial uses should be encouraged for the treatment of diseases caused by MDR *E. coli.* Animal neutering could reduce the risk of canine prostatitis and pyometra which may lead to life-threatening BSIs. Additionally, appropriate hygiene management is considered a preventive intervention to improve pet health.

## Materials and methods

### Ethics approval

This study was conducted according to the faculty regulations and approved by the Institutional Biosafety Committee of Faculty of Veterinary Science, Chulalongkorn University (CU-VET-IBC) (Protocol code IBC 2131030) on 13 December 2020. The authors confirm their adherence to the ethical policies outlined in the journal’s author guidelines. All uterine samples utilised for bacterial isolation in this study were obtained from canine patients who had undergone ovariohysterectomy for therapeutic purposes, under the care of licensed veterinarians in small animal hospitals, with the consent of the owners. The acquisition of samples from animal patients was conducted in compliance with the approval granted by the Institutional Animal Care and Use Committee of the Faculty of Veterinary Science, Chulalongkorn University (Protocol code 2131054) on January 26, 2021.

### Bacterial isolates

A total of 104 *E. coli* from non-urinary extraintestinal infections, including canine pyometra (n = 61), canine PAs (n = 38), and canine and feline BSIs (n = 5) collected from 2016 to 2021, were included for *E. coli* phylogrouping, ExPEC VG detection, and screening of FQ^R^ and 3GC^R^. The 61 *E. coli* strains from pyometra were isolated from the endometrium of 48 out of 100 uteri of female dogs that underwent ovariohysterectomy for surgical treatment of canine pyometra at the Obstetrics, Gynecology, and Reproduction Unit, Small Animal Teaching Hospital, Chulalongkorn University, Thailand. From 12 dogs, two distinct *E. coli* isolates were recovered from the uterus, each presenting different colony characteristics. Additionally, two separate *E. coli* isolates were recovered: one from the endometrium and another from the blood of the same dog suffering from pyometra, which had progressed to sepsis. The 38 isolates from canine PAs were obtained from PA exudate collected by ultrasound-guided aspiration, and the 5 isolates (2 from dogs and 3 from cats) causing BSI were isolated from positive blood culture bottles using the BD BACTEC™ FX blood culture system (Becton-Dickenson, USA).

### *E. coli* phylogrouping and ExPEC virulence gene detection

Phylogroups and ExPEC VG carriage of the 104 *E. coli* were identified using PCR-based methods. Genomic DNA was extracted using Nucleospin^®^ Tissue DNA extraction kits (Machery-Nagel, Germany). Clermont *E. coli* phylo-typing, which consists of a quadruplex PCR panel and two simplex PCRs, was performed to differentiate *E. coli* phylogroup A, B1, B2, C, D, E, F and *E. coli* cryptic clades^35^. VGs associated with extraintestinal pathogenesis of *E. coli*, including adhesion (*afa*, *crl*, *fimA*, *iha*, *papC*, *sfa/foc*, *yfcV*, and *tsh*), cytotoxicity (*hlyA*, *hlyE*, *cnf*, *sat*, and *vat*), iron acquisition (*iroN*, *fyuA*, *irp1*, *chuA*, *iutA*, and *iucD*), and bacterial cell protection (*bssS*, *hmsP*, *iss*, and *kpsII*) were detected by PCR (see Supplementary Table S4 online). All PCRs were conducted in a 25 µL total volume containing 5X Firepol Master Mix (Solis Biodyne, Estonia), and the concentration of each primer was 0.2 µM.

Isolates that tested positive for ≥ 2 out of 5 VGs (*papC*, *sfa/foc*, *afa*, *iutA*, and *kpsII*) were presumably classified as ExPEC, whereas isolates demonstrating ≥ 3 out of 4 VGs (*yfcV*, *vat*, *fyuA*, and *chuA*) were presumably classified as UPEC^4,6^.

### Screening of FQ- and/or 3GC-resistant *E. coli* and antimicrobial susceptibility testing

FQ^R^ and 3GC^R^ were examined in all 104 *E. coli* isolates using ciprofloxacin, cefotaxime, and ceftazidime disk diffusion methods^36^. Isolates displaying resistance to cefotaxime and/or ceftazidime were included for detection of ESBL and AmpC β-lactamase production by the combination disk test and cefoxitin disk diffusion test, respectively. The AMR phenotypes of FQ^R^ and/or 3GC^R^ *E. coli* were determined using broth microdilution with a customised Sensititre™ plate model COMPGN1F (Thermo Scientific, UK). The plate contained amikacin, amoxicillin/clavulanic acid, ampicillin, cefazolin, cefovecin, cefpodoxime, ceftazidime, cephalexin, chloramphenicol, doxycycline, enrofloxacin, gentamicin, imipenem, marbofloxacin, orbifloxacin, piperacillin/tazobactam, pradofloxacin, tetracycline, and sulfamethoxazole/trimethoprim. Resistance to the drugs was interpreted according to the minimum inhibitory concentration (MIC) breakpoints for veterinary isolates from the Clinical and Laboratory Standards Institute^37^.

### Detection of antimicrobial resistance genes

Common ARGs in *E. coli* encompassing β-lactamases (*bla*_OXA_, *bla*_SHV_, and *bla*_TEM_), ESBL (*bla*_CTX-M_ groups), cephalosporinases (ACC, CIT, DHA, EBC, FOX, and MOX groups), PMQR [*aac(6*′*)-Ib-cr*, *qepA*, *qnrA*, *qnrB*, *qnrC*, *qnrD*, and *qnrS*], aminoglycoside-modifying enzymes [*aac(3)-IIa*, *aac(6*′*)-Ib*, *aac(6*′*)-Ib-cr*, *ant(2*′′*)-Ia*, *aph(3*′′*)-Ib*, and *aph(6)-Id*], tetracycline efflux proteins [*tet*(A), *tet*(B), and *tet*(C)], alternative dihydropterate synthase (*sul1* and *sul2*), alternative dihydrofolate reductase (*dfrA1*, *dfrA5*, *dfrA7*, *dfrA12*, *dfrA17*, and *dfrB)*, florfenicol/chloramphenicol efflux protein (*floR*), chloramphenicol efflux protein (*cmlA*), and chloramphenicol acetyltransferase (*catA*) were detected in 44 *E. coli* exhibiting resistance to 3GCs and/or FQs by using PCR-based methods (see Supplementary Table S5 online).

### Strain selection for whole genome sequencing

The clonal relatedness of 44 FQ^R^ and/or 3GC^R^ *E. coli* from pyometra (19 isolates), PAs (20 isolates), and BSIs (5 isolates) was assessed by analysing REP-PCR DNA band patterns with more than 70% similarity^38^. Genetically related *E. coli* strains from different diseases and non-genetically related *E. coli* strains from the same disease, which contained the highest numbers of VGs and ARGs, were selected for WGS. A total of 12 *E. coli* strains, including 4 from pyometra, 5 from PAs, and 3 from BSIs, were chosen. The genomes of seven characterised 3GC^R^ *E. coli* strains associated with global high-risk clones from canine and feline UTIs^12^, including ST38, ST131, ST617, ST641, ST648, and ST1193, were additionally sequenced for comparison. Genomic DNA extraction was performed using the G-spin™ Total DNA Extraction Mini Kit (intron Bio, South Korea) for short-read Illumina sequencing and the Nucleospin® Tissue DNA extraction kits (Machery-Nagel, Germany) for long-read Oxford Nanopore Technologies (ONT) sequencing. DNA libraries were prepared using the NEBNext® Ultra™ DNA Library Prep Kit (New England Biolabs, USA) and were subsequently loaded into an Illumina NextSeq system (Illumina, USA) to obtain 150 bp pair-end reads by a private service company (Celemic, South Korea). For long-read sequencing, the Native Barcoding Kit (SQK-NBD112.24) was utilised for multiplex library preparation from the genomic DNA, followed by loading into an R10.4 flowcell (FLO-MIN112) (ONT, UK). Trimmomatic v.0.39 was employed to trim short reads and remove adaptors to obtain high-quality reads^39^. Guppy v.6.3.8 was used for base-calling and de-multiplexing of long reads. Unicycler pipeline v.0.4.8 was employed for the assembly of short and long reads, resulting in generation of circular chromosomes and plasmids^40^.

### Pan-genome, phylogenetic and bioinformatic analyses

The pan-genome of 19 sequenced strains was analysed to identify core genes and accessory genes using Roary version 3.11.2^41^. The coreSNPs were extracted from core genome alignment using SNP sites for constructing a phylogenetic tree using RAxML version 8 with 1000 bootstraps^42,43^. The coreSNP tree was visualised using Interactive Tree of Life (iTOL) (https://itol.embl.de/itol.cgi). STs and complex types were determined based on Achtman’s MLST and cgMLST, respectively, by submitting short reads to EnteroBase (enterobase.warwick.ac.uk)^44^. A minimum spanning tree based on cgMLST of 2513 core genes was generated using GrapeTree (available at enterobase.warwick.ac.uk) to illustrate genomic relationships among the whole-genome-sequenced strains^45^.

Acquired ARGs and point mutations of QRDR associated with FQ^R^ were detected using ResFinder v.4.1 and the NCBI AMR Finder tool^46,47^. VGs were identified using the Virulence Factor Database (VFDB)^48^. MGEs, including plasmids and PAIs, were identified using PlasmidFinder and IslandViewer 4, respectively^49,50^. The complete genome of UPEC strain CFT073 was utilised as the reference genome for PAI analysis. FimHTyper 1.0 and SerotypeFinder 2.0 were employed to determine *E. coli* FimH types and serotypes, respectively^51,52^. The sequenced contigs were submitted to the NCBI Prokaryotic Genome Annotation Pipeline for ORF prediction and gene annotation.

### Statistical analysis

Fisher’s exact test was used to ascertain the association between various phylogroups and/or disease patterns and the presence of the VGs, and ExPEC and/or UPEC genotypes and disease patterns. The investigation was assessed by using IBM^®^ SPSS^®^ Statistics version 26. Statistical significance was set at *p* < 0.05.

### Supplementary Information

Supplementary Information.

## Data Availability

The genome sequences described in this study were deposited in the NCBI database under BioProject PRJNA914526. All data analysed during this study is included in this published article and its supplementary information. Therefore, all data from this study is available to the public.
