# Supplementary material for: Multidrug-resistant Escherichia coli causing canine pyometra and urinary tract infections are genetically related but distinct from those causing prostatic abscesses
Source: Sci Rep. 2024 May 24;14:11848. doi: 10.1038/s41598-024-62028-9 (PMC11116460; doi:10.1038/s41598-024-62028-9)
Supplement: Supplementary file 1 — Supplementary Information. [file 41598_2024_62028_MOESM1_ESM.pdf]

Supplementary Tables and Figures

Multidrug-resistant *Escherichia coli* causing canine pyometra and urinary tract infections are genetically related but distinct from those causing prostatic abscesses

Parinya Sroithongkham, Naiyaphat Nittayasut, Jitrapa Yindee, Pattaraporn Nimsamer, Sunchai Payungporn, Komkiew Pinpimai, Suppawiwat Ponglowhapan, Pattrarat Chanchaithong

Supplementary Table S1. Number of *Escherichia coli* (n=44) exhibiting fluoroquinolones resistance (FQ^R) and/or third-generation cephalosporin resistance ($3GC^R$)

**Sources* (No.)**	**Resistance phenotype**							**Total**
	**FQ^R, $3GC^S$ (n=26)**	**FQ^S, $3GC^R$ (n=5)**			**FQ^R, $3GC^R$ (n=13)**			
		**ESBL**	**AmpC**	**ESBL+AmpC**	**ESBL**	**AmpC**	**ESBL+AmpC**	
Pyometra (n=19)	12	2	0	0	3	0	2	19
Prostatic abscess (n=20)	12	1	1	0	4	0	2	20
Bloodstream infection (n=5)	2	0	1	0	1	1	0	5
Total (44)	26	3	2	0	8	1	4	44

^{R}, Resistance
^{S}, Susceptible

Supplementary Table S2. Genome characteristics and genetic features of 19 *Escherichia coli* strains subjected to whole-genome sequencing.

Phylo-group	ST (allelic profile) *	Serotype	Strain	Disease	Host	Phenotype **	cgMLST	Chromosomal size (bp)	ARGs on chromosome and QRDR mutation	No. of plasmid	Plasmid (size, replicon); ARGs/ virulence genes	Genbank accession no.
B2	131 (53-40-47-13-36-28-29)	O16:H5	CUVET20-PYO1	Pyometra	Dog	$3GC^R$	191739	5,084,250	*bla*$_{CTX-M-27;}$ *gyrA*:pS83L, *parE*:pI529L	1	**pCUVET21-PYO1.1** (146,827 bp, IncFIB/Col156/Q1/FII); *bla*$_{TEM-}$1B, *aadA5*, *aac(3)-IId*, *aph(3'')-Ib*, *aph(6)-Id*, *tet*(A), *dfrA17*, *sul1*, *sul2*, *mph*(A), *qacE* / *senB*	CP115390-CP115391
		O25:H4	CUVET17-1136	Urinary tract infection	Dog	$3GC^R$, FQ^R	222870	5,289,576	*bla*$_{CTX-M-15}$, *bla*$_{OXA-1}$, *aac(6')-Ib-cr*, *aac(3)-IIa*, *catB3* ; *gyrA*:pS83L, *gyrA*:pD87N, *parC*:pS80I, *parC*:pE84V, *parE*:pI529L	1	**pCUVET17-1136.1 (**146,752 bp, IncFIB/FIA/FIC(FII)/Col156); *tet*(A) / *iutA, iucD, iucC, iucB, iucA*	CP115324-CP115325
	1193 (14-14-10-200-17-7-10)	O18:H5	CUVET20-PYO2	Pyometra	Dog	FQ^R	218675	5,119,498	*gyrA*:pS83L, *gyrA*:pD87N, *parC*:pS80I, *parE*:pL416F	3	**pCUVET20-PYO2.1** (100,876 bp, IncFIB/Col156/FIA); *bla*$_{TEM-1B}$, *aadA5*, *aac(3)-IId*, *aph(6)-Id*, *aph(3'')-Ib*, *tet*(A), *dfrA17*, *sul1*, , *sul2*, *mph*(A), *qacE/ senB*<br>**pCUVET20-PYO2.2** (6,072 bp, Col156); -<br>**pCUVET20-PYO2.3** (2,104 bp, ColBS512); -	CP115386-CP115389
		O18:H5	CUVET21-H2	Bloodstream infection	Dog	FQ^R	212951	5,124,677	*gyrA*:pS83L, *gyrA*:pD87N, *parC*:pS80I, *parE*:pL416F	6	**pCUVET2-H2.1** (84,550 bp, IncFIB/Col156/FIA); *bla*$_{TEM-1B}$, *aac(3)-IId/ senB*<br>**pCUVET2-H2.2** (5,570 bp, ColRNAI); -<br>**pCUVET2-H2.3** (5,165 bp, Col156); -<br>**pCUVET2-H2.4** (2,113 bp, ColBS512); -<br>**pCUVET2-H2.5** (1,989 bp, ColpVC); -<br>**pCUVET2-H2.6** (1,552 bp, ColMG828); -	CP115343-CP115350

Phylo-group	ST (allelic profile) *	Serotype	Strain	Disease	Host	Phenotype **	cgMLST	Chromosomal size (bp)	ARGs on chromosome and QRDR mutation	No. of plasmid	Plasmid (size, replicon); ARGs/ virulence genes	Genbank accession no.
		O75:H5	CUVET18-565	Urinary tract infection	Dog	3GC^R, FQ^R	222758	4,990,306	*gyrA*:pS83L, *gyrA*:pD87N, *parC*:pS80I, *parE*:pL416F	3	**pCUVET18-565.1 (**98,809 bp, IncFIB/Col156/FIA); *bla*$_{CTX-M-27}$, *aadA5*, *aph(3'')-Ib*, *aph(6)-Id*, *tet*(A), *dfrA17*, *sul1*, *sul2*, *mph*(A), *qacE*/ *senB*<br>**pCUVET18-565.2** (4,548 bp, pEC931); -<br>**pCUVET18-565.3** (2,113 bp, ColBS512); -	CP115320-CP115323
	12 (13-13-9-13-16-10-9)	O4:H5	CUVET16-707	Prostatic abscess	Dog	3GC^R, FQ^R	222761	5,043,683	*bla*$_{CTX-M-15}$, *aadA16*, *aac(6')-Ib-cr*, *aac(3)-IId*, *aac(6)-Ia*, *ant(3'')-IIa*, *tet*(A), *dfrA27*, *sul1*, *sul1*, *catA2*, *cmlA*, *qacE*	1	**pCUVET16-707.1** (5,462 bp, no Inc); *qnrB6*, *sul1*, *qacE*	CP115356-CP115357
	998 (13-52-156-14-17-25-17)	O50:H6	CUVET21-PYO5	Pyometra	Dog	3GC^R	222869	5,274,841	*bla*$_{CTX-M-14,}$ *mph*(A); *gyrA*:pS83L	2	**pCUVET21-PYO5.1 (**40,929 bp, IncX1**)**<br>**pCUVET21-PYO5.2 (**4,671 bp, ColRNAI**)**	CP116948-CP116950
F	648 (92-4-87-96-70-58-2)	O1:H6	CUVET20-PYO4	Pyometra	Dog	3GC^R, FQ^R	218671	5,199,531	*gyrA*:pS83L, *gyrA*:pD87N, *parC*:pS80I, *parE*:pS458A	4	**pCUVET20-PYO3.1** (127,633 bp, IncFIB/FII/FIA); *bla*$_{TEM-1B}$, *bla*$_{OXA-1}$, *aadA5*, *aac(6')-Ib-cr*, *aac(3)-IId*, *dfrA17*, *sul1*, *catB3*, *mph*(A), *mph*(A)/ *iutA, iucD, iucC, iucB, iucA*<br>**pCUVET20-PYO3.2** (111,925 bp, IncFIB); *bla*$_{CTX-M-15}$<br>**pCUVET20-PYO3.3** (89,033 bp, *repB* plasmid); -<br>**pCUVET20-PYO3.4** (4,082 bp, Col8282); -	CP115376-CP115380
			CUVET17-1010	Urinary tract infection	Dog	3GC^R, FQ^R	222763	5,353,451	*bla*$_{CTX-M-15}$, *bla*$_{OXA-1}$, *aac(6')-Ib-cr*, *aac(3)-IIa*, *tet*(A); *gyrA*:pS83L, *gyrA*:pD87N, *parC*:pS80I, *parE*:pS458A	3	**pCUVET17-1010.1** (134,505 bp, IncFIB/FII/FIA); *aadA5*, *tet*(B), *dfrA17*, *sul1*, *catA1*, *mph*(A), *qacE*/ *iutA, iucD, iucC, iucB, iucA*<br>**pCUVET17-1010.2** (93,950 bp, p0111); -<br>**pCUVET17-1010.3** (2,080 bp, ColBS512); -	CP115326-CP115329

Phylo-group	ST (allelic profile) *	Serotype	Strain	Disease	Host	Phenotype **	cgMLST	Chromosomal size (bp)	ARGs on chromosome and QRDR mutation	No. of plasmid	Plasmid (size, replicon); ARGs/ virulence genes	Genbank accession no.
			CUVET19-1637	Urinary tract infection	Dog	$3GC^R$, FQ^R	223830	5,158,526	*gyrA*:pS83L, *gyrA*:pD87N, *parC*:pS80L, *parC*:pE84K	5	**pCUVET19-1637.1** (139,825 bp, IncFIB/FII/FIA); *bla_{OXA-1}*, *aadA5*, *aac(6')-Ib-cr*, *tet*(B), *dfrA17*, *sul1*, *catA1*, *catB3*, *mph*(A), *anr*, *qacE*, / *iutA*, *iucD*, *iucC*, *iucB*, *iucA*<br>**pCUVET19-1637.2** (52,617 bp, IncI1); *$bla_{CMY-148}$*<br>**pCUVET19-1637.3** (3,022 bp, CP070076); -<br>**pCUVET19-1637.4** (2,093 bp, ColBS512); -<br>**pCUVET19-1637.5** (1,277 bp, ColMG828); -	CP121673-CP121678
		O83:42	CUVET21-1783	Bloodstream infection	Dog	$3GC^R$, FQ^R	222867	5,119,320	*bla_{CMY-2}*, *sul2*, *floR*; *gyrA*:pS83L, *gyrA*:pD87N, *parC*:pS80I, *parE*:pS458A	0	-	CP115342
	457 (101-88-97-108-26-79-2)	O11:H5	CUVET19-1704	Prostatic abscess	Dog	FQ^R	218676	4,953,103	*gyrA*:pS83L, *gyrA*:pD87Y, *parC*:pS80I, *parE*:pS458A	2	**pCUVET19-1704.1** (149,951 bp, IncFIB/FIC(FII)/FIA); *bla_{TEM-1B}*, *aadA2*, *aac(3)-IId*, *ant(3'')-Ia*, *tet*(A), *tet*(M), *dfrA12*, *sul2*, *cmlA1*, *floR*, *qacL*, *lnu*(F), / *iutA*, *iucD*, *iucC*, *iucB*, *iucA*<br>**pCUVET19-1704.2** (16,660 bp, IncX1); -	CP115358-CP115360
A	44 (10-11-4-8-8-8-7)	O101:H4	CUVET17-972	Prostatic abscess	Dog	$3GC^R$, FQ^R	222759	4,868,421	*aph(3'')-Ib*, *aph(6)-Id*, *tet*(A), *sul2*, *floR*; *gyrA*:pS83L, *gyrA*:pD87N, *parC*:pS80I, *parE*:pS458T	5	**pCUVET17-972.1** (138,366 bp, IncFIB/FIC(FII)/FIA); *$bla_{CTX-M-15}$*, *bla_{OXA-1}*, *aadA5*, *aac(6')-Ib-cr*, *aac(3)-IIa*, *dfrA17*, *sul1*, *mph(A)*, *qacE* / *iutA*, *iucD*, *iucC*, *iucB*, *iucA*<br>**pCUVET17-972.2** (12,602 bp, Col440I/ Col440I); -<br>**pCUVET17-972.3** (6,648 bp, ColRNAI); -<br>**pCUVET17-972.4** (3,133 bp, Col440I); -	CP115370-CP115375

Phylo-group	ST (allelic profile) *	Serotype	Strain	Disease	Host	Phenotype **	cgMLST	Chromosomal size (bp)	ARGs on chromosome and QRDR mutation	No. of plasmid	Plasmid (size, replicon); ARGs/ virulence genes	Genbank accession no.
											**pCUVET17-972.5** (1,551 bp, ColMG828); -	
	617 (10-11-4-8-8-13-73)	O101:H9	CUVET16-242	Urinary tract infection	Dog	3GC^R, FQ^R	222764	5,199,561	*bla*$_{CTX-M-14}$; *gyrA*:pS83L, *gyrA*:pD87N, *parC*:pS80I, *parE*:pS458A	8	**pCUVET16-242.1** (145,549 bp, IncR/FIC(FII)); *bla*$_{TEM-1B}$, *aadA2*, *aac(3)-IId*, *ant(3")-Ia*, *tet*(A), *tet*(M), *dfrA12*, *sul3*, *cmlA*, *qacL* **pCUVET16-242.2** (92,087 bp, IncFIB,/FIC(FII)); *iutA*, *iucD*, *iucC*, *iucB*, *iucA* **pCUVET16-242.3** (43,540 bp, IncX9/X1); - **pCUVET16-242.4** (3,811 bp, Col8282); - **pCUVET16-242.5** (4,021 bp, ColRNAI); - **pCUVET16-242.6** (3,231 bp, ColRNAI); - **pCUVET16-242.7** (2,372 bp, p4_005215); - **pCUVET16-242.8** (1,436 bp, ColMG828); -	CP115333-CP115341
	744 (10-11-135-8-8-8-2)	O101:H9	CUVET20-1667	Prostatic abscess	Dog	3GC^R, FQ^R	218673	4,757,980	*gyrA*:pS83L, *gyrA*:pD87N, *gyrA*:pA56T, *parC*:pS80I	3	**pCUVET20-1667.1** (137,735 bp, IncFIB/FIC(FII)); *bla*$_{CTX-M-55}$, *ant(3")-Ia*, *ant(3")-Ia*, *aac(3)-IIa*, *aph(3')-Ia*, *tet*(A), *sul3*, *floR*, *lnu*(F), *qacL*/ *iutA*, *iucD*, *iucC*, *iucB*, *iucA* **pCUVET20-1667.2** (6,478 bp, IncQ1); - **pCUVET20-1667.3** (3,603 bp, ColRNAI); -	CP115361-CP115364
	13037 (9-41-1-8-8-8-6)	O9:H4	CUVET19-1426	Prostatic abscess	Dog	3GC^R, FQ^R	191738	4,593,032	*gyrA*:pS83L, *gyrA*:pD87N, *parC*:pS80I	4	**pCUVET19-1426.1** (123,620 bp, IncR/N); *bla*$_{CTX-M-55}$, *bla*$_{TEM-1B}$, *bla*$_{TEM-1B,}$ *qnrS1*, *aadA1*, *aadA2*, *tet*(A), *dfrA12*, *sul2*, *sul3*, *floR*, *cmlA*, *qacL*, *mef*(B) **pCUVET19-1426.2** (69,932 bp, IncFII); *bla*$_{TEM-1B,}$ *rmtB* **pCUVET19-1426.3** (43,410	CP115365-CP115369

Phylo-group	ST (allelic profile) *	Serotype	Strain	Disease	Host	Phenotype **	cgMLST	Chromosomal size (bp)	ARGs on chromosome and QRDR mutation	No. of plasmid	Plasmid (size, replicon); ARGs/ virulence genes	Genbank accession no.
											bp, IncX1); -<br>**pCUVET19-1426.4** (6,710 bp, Col156); -	
B1	101 (43-41-15-18-11-7-6)	O55:H10	CUVET21-37	Bloodstream infection	Cat	$3GC^R$, FQ^R	222762	4,790,718	*gyrA*:pS83L	4	**pCUVET21-37.1** (141,537 bp, IncFIB/FIC(FII)/FIA); *bla*$_{\text{TEM-1B,}}$ *aph(3'')-Ib*, *aph(6)-Id*, *dfrA14*, *sul2*, / *iroB*, *iroC*, *iroD*, *iroE*, *iroN*, *iutA*, *iucD*, *iucC*, *iucB*,*iucA*<br>**pCUVET21-37.2** (76,741 bp, IncFII); *bla*$_{\text{CTX-M-55}}$, *qnrS1*, tet(M), *floR*<br>**pCUVET21-37.3** (45,497 bp, IncX1); -<br>**pCUVET21-37.4** (1,516 bp, ColMG828); -	CP115351-CP115355
	641 (9-6-33-131-24-8-7)	O87:H10	CUVET18-789	Urinary tract infection	Cat	$3GC^R$, FQ^R	222868	4,689,626	*tet*(A); *gyrA*:pS83L, *gyrA*:pD87N, *parC*:pS80I	7	**pCUVET18-789.1** (113,740 bp, IncFIB); -<br>**pCUVET18-789.2** (96,320 bp, IncFIB/FII); *bla*$_{\text{CTX-M-55}}$, *qnrS1*, *aac(3)-IId*, *catA2*, *mph*(A), *erm*(B)<br>**pCUVET18-789.3** (48,816 bp, IncN); *bla*$_{\text{TEM-1B}}$, *qnrS1*<br>**pCUVET18-789.4** (10,121 bp, IncX1); -<br>**pCUVET18-789.5** (6,477 bp, IncQ1); -<br>**pCUVET18-789.6** (4,663 bp, Col440I); -<br>**pCUVET18-789.7** (2,464 bp, CP072459); -	CP115312-CP115319
D	38 (4-26-2-25-5-5-19)	O1:H15	CUVET16-394	Urinary tract infection	Cat	$3GC^R$, FQ^R	222871	5,101,217	*gyrA*:pS83L, *gyrA*:pD87N, *parC*:pS80I	2	**pCUVET16-394.1** (157,054 bp, IncFIB/Col156/FII/FIA); *bla*$_{\text{CTX-M-27}}$, *aadA5*, *aph(3'')-Ib*, *aph(6)-Id*, *tet*(A), *dfrA17*, *sul1*, *sul2*, *mph(A)*, *erm*(B), *qacE*, / *senB*<br>**pCUVET16-394.2** (5,164 bp, Col156); -	CP115330-CP115332

* Allelic profile, (*adk-fumC-gyrB-icd-mdh-purA-recA*)

** $3GC^R$, third-generation cephalosporin resistance; and FQ^R, fluoroquinolone resistance

Supplementary Table S3. Pathogenicity islands identified in 19 sequenced *Escherichia coli* strains isolated from extraintestinal infections in dogs and cats.

Phylo-group	ST	Strain	Disease *	PAI name	insertion site	Size (kb)	Virulence factor	Position
B2	131	CUVET20-PYO1	PYO	PAI-CUVET20-PYO1-pheV	*pheV*	104	Pyelonephritis-associated pili, iron-regulated gene homologue adhesin (Iha), secreted autotransporter toxin (Sat), aerobactin, antigen 43 and group 2 capsule	800,969-904,916
				PAI-CUVET20-PYO1-asnT	*asnT*	40	Yersiniabactin	2,095,672-2,136,419
				PAI-CUVET20-PYO1-pheU	*pheU*	72	Pyelonephritis-associated pili, cytotoxic necrotising factor (CNF1), α-hemolysin, and contact-dependent growth inhibitor (CdiB)	4,421,681-4,493,930
		CUVET17-1136	UTI	PAI-CUVET17-1136-asnT	*asnT*	40	Yersiniabactin	772,493-812461
				PAI-CUVET17-1136-pheV	*pheV*	94	Pyelonephritis-associated pili, Iha, Sat, aerobactin, antigen 43, and group 2 capsule	1,891,707-1,986,231
				PAI-CUVET17-1136-pheU	*pheU*	70	Pyelonephritis-associated pili, CNF1, α-Hemolysin, and CdiB	3,348,822-3,418,529
				PAI-CUVET17-1136-icd	*icd*	50	Increase serum survival protein, Sit system	5,081,931-5,131,540
	1193	CUVET20-PYO2	PYO	PAI-CUVET20-PYO2-asnT	*asnT*	40	Yersiniabactin	2,805,229-2,845,192
				PAI-CUVET20-PYO2-pheV	*pheV*	60	Pyelonephritis-associated pili, Iha, Sat, aerobactin, and group 2 capsule	3,991,628-4,051,628
		CUVET21-H2	BSI	PAI-CUVET21-H2-leuX	*leuX*	59	Fec system and antigen 43	511-973 570,700
				PAI-CUVET21-H2-asnT	*asnT*	40	Yersiniabactin	2,775,428-2,815,393
				PAI-CUVET21-H2-pheV	*pheV*	57	Pyelonephritis-associated pili, Iha, Sat, aerobactin and group 2 capsule	4,008,817-4,066,211
		CUVET18-565	UTI	PAI-CUVET18-565-leuX	*leuX*	49	Fec system and antigen 43	613,485-661,278
				PAI-CUVET18-565-thrW	*thrW*	9	Vacuolating autotransporter toxin (Vat)	1,015,029-1,024,114
				PAI-CUVET18-565-asnT	*asn*	40	Yersiniabactin	2,759,581-2,799,554
				PAI-CUVET18-565-pheV	*pheV*	60	Pyelonephritis-associated pili, Iha, Sat, aerobactin and group 2 capsule	3,873,633-3,933,642
	12	CUVET16-707	PA	PAI-CUVET16-707-pheV	*pheV*	22	Pyelonephritis-associated pili	874,678-894,881
				PAI-CUVET16-707-asnT	*asnT*	40	Yersiniabactin	2,015,436-2,055,395
				PAI-CUVET16-707-serX	*serX*	87	Antigen 43, S fimbriae, salmochelin, and CdiA	2,949,666-3,036,637
				PAI-CUVET16-707-leuX	*leuX*	71	Pyelonephritis-associated pili, α-hemolysin and CNF1	4,304,799-4,369,787
	998	CUVET21-PYO5	PYO	PAI-CUVET21-PYO5-sec	*sec*	73	α-hemolysin	44,607-118,119
				PAI-CUVET21-PYO5-serX	*serX*	63	S fimbriae, F1C fimbriae and salmochelin	2,076,881-2,140,319

**Phylo-group**	**ST**	**Strain**	**Disease ***	**PAI name**	**insertion site**	**Size (kb)**	**Virulence factor**	**Position**
				PAI-CUVET21-PYO5-asnT	*asnT*	40	Yersiniabactin	3,114,486-3,154,451
				PAI-CUVET21-PYO5-pheU	*pheU*	36	S fimbriae and F1C fimbriae	4,516,107-4,551,662
				PAI-CUVET21-PYO5-leuX	*leuX*	37	CNF1, α-hemolysin, and Fec system	4,690,212-4,727,697
F	648	CUVET20-PYO4	PYO	PAI-CUVET20-PYO4-asnT	*asnT*	40	Yersiniabactin	1,182,793-1,222,761
				PAI-CUVET20-PYO4-sec	*sec*	49	Afimbrial adhesin and antigen 43	3,154,900-3,203,759
		CUVET17-1010	UTI	PAI-CUVET17-1010-asnT	*asnT*	40	Yersiniabactin	2,128,594-2,168,562
				PAI-CUVET17-1010-arg	*arg*	7.4	Increase serum survival protein	2,914,575-2921,991
				PAI-CUVET17-1010-fecI	*fecI*	7	Antigen 43	4,492,072-4,499,089
				PAI-CUVET17-1010-pheU	*pheU*	75	Pyelonephritis-associated pili, CNF1, α-hemolysin, and CdiB	4,660,034-4,735,786
		CUVET19-1637	UTI	PAI-CUVET19-1637-asnT	*asnT*	40	Yersiniabactin	2,039,104-2,078,994
	457	CUVET19-1704	PA	PAI-CUVET19-1704-arg	*arg*	47	Increase serum survival protein	3,538,218-3,585,048
A	617	CUVET16-242	UTI	PAI-CUVET16-242-asnT	*asnT*	64	Yersiniabactin and type IV secretion system	1,874,740-1,939,346
				PAI-CUVET16-242-pheU	*pheU*	73	S fimbriae and F1C fimbriae	4,425,263-4,498,632
	13037	CUVET19-1426	PA	PAI-CUVET19-1426-arg	arg	13	Increase serum survival protein, ompT	3,259,294-3,272,176
B1	101	CUVET21-37	BSI	PAI-CUVET21-37-asnT	*asnT*	43	Yersiniabactin	1,850,606-1,893,282
				PAI-CUVET21-37-serX	*serX*	49	Salmochelin	2,830,617-2,879,227
				PAI-CUVET21-37-arg	*arg*	15	Increase serum survival protein, ompT	3,423,790-3,439,007
	641	CUVET18-789	UTI	PAI-CUVET18-789-arg	arg	11	Increase serum survival protein, ompT	3,350,222-3,361,790
D	38	CUVET16-394	UTI	PAI-CUVET16-394-asnT	*asnT*	40	Yersiniabactin	201,518-241,487
				PAI-CUVET16-394-arg	*arg*	23	Increase serum survival protein	954,712-977,990
				PAI-CUVET16-394-icd	*icd*	21	Increase serum survival protein	1,577,987-1,599,029
				PAI-CUVET16-394-pheV	*pheV*	119	Pyelonephritis-associated pili, Iha, Sat, aerobactin, antigen 43 and group 2 capsule	4,167,029-4,168,072

*, PYO, pyometra; PA, prostatic abscesses; BSI, bloodstream infections; and UTI, urinary tract infections

Supplementary Table S4. Primers for detection of virulence genes in extraintestinal pathogenic *Escherichia coli*.

Gene	Primer name	Oligonucleotide sequence (5′–3′)	Annealing temperature (°C)	Amplicon size (bp)	Reference
**Multiplex I**					
hlyA	hlyA-f	GTCCATTGCCGATAAGTTT	58	352	[1]
	hlyA-r	AAGTAATTTTTGCCGTGTTTT			
iha	iha-f	TAGTGCGTTGGGTTATCGCTC	58	609	[1]
	iha-r	AAGCCAGAGTGGTTATTCGC			
afa	afa-f	TAAGGAAGTGAAGGAGCGTG	58	810	[1]
	afa-r	CCAGTAACTGTCCGTGACA			
sfa/foc	sfa-f	GTCCTGACTCATCTGAAACTGCA	58	1242	[1]
	sfa-r	CGGAGAACTGGGTGCATCTTA			
**Multiplex II**					
crl	crl-f	TTTCGATTGTCTGGCTGTATG	58	250	[2]
	crl-r	CTTCAGATTCAGCGTCGTC			
cnf	cnf-f	TCGTTATAAAATCAAACAGTG	58	446	[1]
	cnf-r	CTTTACAATATTGACATGCTG			
sat	sat-f	TGCTGGCTCTGGAGGAAC	58	667	[1]
	sat-r	TTGAACATTCAGAGTACCGGG			
iroN	iroN-f	ATCCTCTGGTCGCTAACTG	58	847	[1]
	iroN-r	CTGCACTGGAAGAACTGTTCT			
**Multiplex III**					
papC	papC-f	TGATATCACGCAGTCAGTAGC	58	501	[3]
	papC-r	CCGGCCATATTCACATAAC			
iucD	iucD-f	ACAAAAAGTTCTATCGCTTCC	58	714	[3]
	iucD-r	CCTGATCCAGATGATGCTC			
tsh	tsh-f	ACTATTCTCTGCAGGAAGTC	58	824	[1]
	tsh-r	CTTCCGATGTTCTGAACGT			
**Multiplex IV**					
vat	vat_for	TCAGGACACGTTCAGGCATTCAGT	63	1100	[4]
	vat_rev	GGCCAGAACATTTGCTCCCTTGTT			
fyuA	fyuA_for	GTAAACAATCTTCCCGCTCGGCAT	63	850	[4]
	fyuA_rev	TGACGATTAACGAACCGGAAGGGA			
chuA	chuA_for	CTGAAACCATGACCGTTACG	63	652	[5]
	chuA_rev	TTGTAGTAACGCACTAAACC			
yfcV	yfcV_for	ACATGGAGACCACGTTCACC	63	292	[6]
	yfcV_rev	GTAATCTGGAATGTGGTCAGG			
**Simplex PCR**					
hlyE	hlyE-F	CCAGAAAGGCATTCTCATTAAG	50	263	[7]
	hlyE-R	CATAATCAACGTAGAATCTGGCT			
fimA	fimA-F	CTCTGGCAATCGTTGTTCTG	50	480	[7]
	fimA-R	CACCAATGGCATAATAACGC			
iutA	iutA_for	GGCTGGACATCATGGGAACTGG	63	302	[8]

Gene	Primer name	Oligonucleotide sequence (5′ –3′)	Annealing temperature (°C)	Amplicon size (bp)	Reference
	iutA_rev	CGTCGGGAACGGGTAGAATCG			
irp1	irp1-F	GGCAGCGAACGTGATTCAC	55	605	[7]
	irp1-R	CAGCAGGATGACGGGCTTC			
iss	iss-F	ATCACATAGGATTCTGCCG	50	300	[7]
	iss-R	CAGCGGAGTATAGATGCCA			
bssS	bssS-F	GTCATTCAGACTCATCCGCTC	55	232	[7]
	bssS-R	GACGCCGATACTCGTTTACC			
hmsP	hmsP-F	CTATCTGGTGCTACAGGCGG	55	520	[7]
	hmsP-R	GCGTGGCGACAGTACCG			
kpsII	kpsII-F	GCGCATTTGCTGATACTGTTG	58	577	[9]
	K2-R	AGGTAGTTCAGACTCACACCT			

Supplementary Table S5. Primers for detection of antimicrobial resistance genes in third-generation cephalosporin and/or fluoroquinolone-resistant *Escherichia coli.*

Gene	Primer name	Oligonucleotide sequence (5′ –3′)	Annealing temperature (°C)	Amplicon size (bp)	Reference
**Simplex PCR**					
ac(6')-Ib-cr	aac(6′)-Ib_For	TTGCGATGCTCTATGAGTGGCTA	55	482	[10]
	aac(6′)-Ib_Rev	CTCGAATGCCTGGCGTGTTT			
aac(6')-Im	aac6′Im-F	CTTGATGCTGGAAATGAATCATG	57	410	This study
	aac6′Im-R	CCAACGATCATATAACCACATTTC			
catA	CatA_F	GCGAAGAAGTTGTCCAT	52	365	[11]
	CatB_ R	ACCGTAAAGAAAAATAAGCAC			
cmlA	cmlA_F	CCGCCACGGTGTTGTTGTTATC	60	698	[12]
	cmlA_R	CACCTTGCCTGCCCATCATTAG			
floR	floR-F	TATCTCCCTGTCGTTCCAG	50	399	[12]
	floR-F	AGAACTCGCCGATCAATG			
qepA	QEPfor	TGGTCTACGCCATGGACCTCA	56	1,137	[13]
	QEPrev	TGAATTCGGACACCGTCTCCG			
qnrA	QnrAm-F	AGAGGATTTCTCACGCCAGG	54	580	[14]
	qnrA_R	GCCATACCTACGGCGATACC			
qnrB	qnrB_F	GATCGTGAAAGCCAGAAAGG	54	476	[15]
	qnrB_R	ATGAGCAACGATGCCTGGTA			
qnrC	qnrC-F	GGGTTGTACATTTATTGAATC	50	447	[16]
	qnrC-R	TCCACTTTACGAGGTTCT			
qnrD	qnrD-F	CGAGATCAATTTACGGGGAATA	54	582	[17]
	qnrD-R	AACAAGCTGAAGCGCCTG			
qnrS	QnrSm-F	GCAAGTTCATTGAACAGGGT	54	428	[14]
	QnrSm-R	TCTAAACCGTCGAGTTCGGCG			
strAB	strAB-F	GCTCAAAGGTCGAGGTGT	54	985	This study
	strAB-R	GTTCCTCAGATGCGGCATA			
sul1	sul1_for	GTGACGGTGTTCGGCATTCT	68	779	[18]
	sul1_rev	TCCGAGAAGGTGATTGCGCT			
sul2	sul2_for	CGGCATCGTCAACATAACCT	66	721	[18]
	sul2_rev	TGTGCGGATGAAGTCAGCTC			
tet(A)	tetA_for	GGCCTCAATTTCCTGACG	55	372	[19]
	tetA_rev	AAGCAGGATGTAGCCTGTGC			
tet(B)	tetB_for	GAGACGCAATCGAATTCGG	55	228	[19
	tetB_rev	TTTAGTGGCTATTCTTCCTGCC			
tet(C)	tetC_for	TGCTCAACGGCCTCAACC	58	379	[19]
	tetC_rev	AGCAAGACGTAGCCCAGCG			
dfrB	dfrB_F	GATCGCCTGCGCAAGAAATC	60	141	[20]
	dfrB_R	AAGCGCAGCCACAGGATAAAT			
**Multiplex I: TEM, SHV and OXA-1-like**					
TEM variants including TEM-1 and TEM-2	MultiTSO-T_for	CATTTCCGTGTCGCCCTTATTC	60	800	[21]
	MultiTSO-T_rev	CGTTCATCCATAGTTGCCTGAC			

Gene	Primer name	Oligonucleotide sequence (5′ –3′)	Annealing temperature (°C)	Amplicon size (bp)	Reference
SHV variants including SHV-1	MultiTSO-S_for	AGCCGCTTGAGCAAATTAAAC	60	713	[21]
	MultiTSO-S_rev	ATCCCGCAGATAAATCACCAC			
OXA-1, OXA-4 and OXA-30	MultiTSO-O_for	GGCACCAGATTCAACTTTCAAG	60	564	[21]
	MultiTSO-O_rev	GACCCCAAGTTTCCTGTAAGTG			
**Multiplex II: CTX-M group 1, group 2 and group 9**					
Variants of CTX-M group 1 including CTX-M-1, CTX-M-3 and CTX-M-15	MultiCTXMGp1_for	TTAGGAARTGTGCCGCTGYA	60	688	[21]
	MultiCTXMGp1-2_rev	CGATATCGTTGGTGGTRCCAT			
Variants of CTX-M group 2 including CTX-M-2	MultiCTXMGp2_for	CGTTAACGGCACGATGAC	60	404	[21]
	MultiCTXMGp1-2_rev	CGATATCGTTGGTGGTRCCAT			
Variants of CTX-M group 9 including CTX-M-9 and CTX-M-14	MultiCTXMGp9_for	TCAAGCCTGCCGATCTGGT	60	561	[21]
	MultiCTXMGp9_rev	TGATTCTCGCCGCTGAAG			
**Multiplex III ACC, FOX, MOX, DHA, CIT and EBC**					
ACC-1 and ACC-2	MultiCaseACC_for	CACCTCCAGCGACTTGTTAC	60	346	[21]
	MultiCaseACC_rev	GTTAGCCAGCATCACGATCC			
FOX-1 to FOX-5	MultiCaseFOX_for	CTACAGTGCGGGTGGTTT	60	162	[21]
	MultiCaseFOX_rev	CTATTTGCGGCCAGGTGA			
MOX-1, MOX-2, CMY-1, CMY-8 to CMY-11 and CMY-19	MultiCaseMOX_for	GCAACAACGACAATCCATCCT	60	895	[21]
	MultiCaseMOX_rev	GGGATAGGCGTAACTCTCCCAA			
DHA-1 and DHA-2	MultiCaseDHA_for	TGATGGCACAGCAGGATATTC	60	997	[21]
	MultiCaseDHA_rev	GCTTTGACTCTTTCGGTATTCG			
LAT-1 to LAT-3, BIL-1, CMY-2 to CMY-7, CMY-12 to CMY-18 and CMY-21 to CMY-23	MultiCaseCIT_for	CGAAGAGGCAATGACCAGAC	60	538	[21]
	MultiCaseCIT_rev	ACGGACAGGGTTAGGATAGY[b]			
ACT-1 and MIR-1	MultiCaseEBC_for	CGGTAAAGCCGATGTTGCG	60	683	[21]
	MultiCaseEBC_rev	AGCCTAACCCCTGATACA			
**Multiplex IV**					
dfrA1	dfr1_f	TGGTAGCTATATCGAAGAATGGAGT	60	425	[22]
	dfr1_r	TATGTTAGAGGCGAAGTCTTGGGTA			
dfrA5	dfr5_f	AGCTACTCTTTAAAGCCTTGACGTA	60	341	[22]
	dfr5_r	TATGTTAGAGGCGAAGTCTTGGGTA			
dfrA7& dfrA17	dfr7&17-f	ACATTTGACTCTATGGGTGTTCTTC	60	280	[22]
	dfr7&17-r	AAAACTGTTCAAAAACCAAATTGAA			
dfrA7	dfr7-r	ACCTCAACGTGAACAGTAGACAAAT	60	227 with dfr7&17-f	[22]
dfrA17	dfr17-r	TCTCTGGCGGGGGTCAAATCTAT	60	171 with	[22]

Gene	Primer name	Oligonucleotide sequence (5′ –3′)	Annealing temperature (°C)	Amplicon size (bp)	Reference
				dfr7&17-f	
dfrA12	dfr12-f	GAGCTGAGATATACACTCTGGCACT	60	155	[22]
	dfr12-r	GTACGGAATTACAGCTTGAATGGT			
**Multiplex V**					
aac(3)-Ia	1a-aac(3)-Ia-F	GACATAAGCCTGTTCGGTT	49	313	[23]
	1a-aac(3)-Ia-R	CCCGCTTTCTCGTAGCA			
aac(3)-IIa	1b-aac(3)-IIa-F	ATGCATACGCGGAAGGC	49	822	[23]
	1b-aac(3)-IIa-R	TGCTGGCACGATCGGAG			
aac(6')-Ih	1c-aac(6′)-Ih-F	TGCCGATATCTGAATC	49	407	[23]
	1c-aac(6′)-Ih-R	ACACCACACGTTCAG0			
**Multiplex VI**					
aph(3')-VI	2a-aph(3′)-VI-F	CGGAAACAGCGTTTTAGA	49	716	[23]
	2a-aph(3′)-VI-R	TTCCTTTTGTCAGGTC			
ant(2")-Ia	2b-ant(2″)-Ia-F	ATCTGCCGCTCTGGAT	49	404	[23]
	2b-ant(2″)-Ia-R	CGAGCCTGTAGGACT			
**Multiplex VII**					
aph(3')-Ia	3a-aph(3′)-Ia-F	CGAGCATCAAATGAAACTGC	54	623	[23]
	3a-aph(3′)-Ia-R	GCGTTGCCAATGATGTTACAG			
aac(6')-Ib	3b-aac(6′)-Ib-F	TATGAGTGGCTAAATCGAT	54	395	[23]
	3b-aac(6′)-Ib-R	CCCGCTTTCTCGTAGCA			
**Primer for full length *bla*$_{\mathbf{CTX\text{-}M}}$ group 1 and 9 detection and sequencing**					
CTX-M group 1	CTX-M-1full_for	CTTCCAGAATAAGGAATCCC	50	949	[24]
	CTX-M-1full_rev	CGTCTAAGGCGATAAACAAA			
CTX-M group 9	CTX-M-9full_for	TGACCGTATTGGGAGTTTG	50	902	[24]
	CTX-M-9full_rev	ACCAGTTACAGCCCTTCG			

a

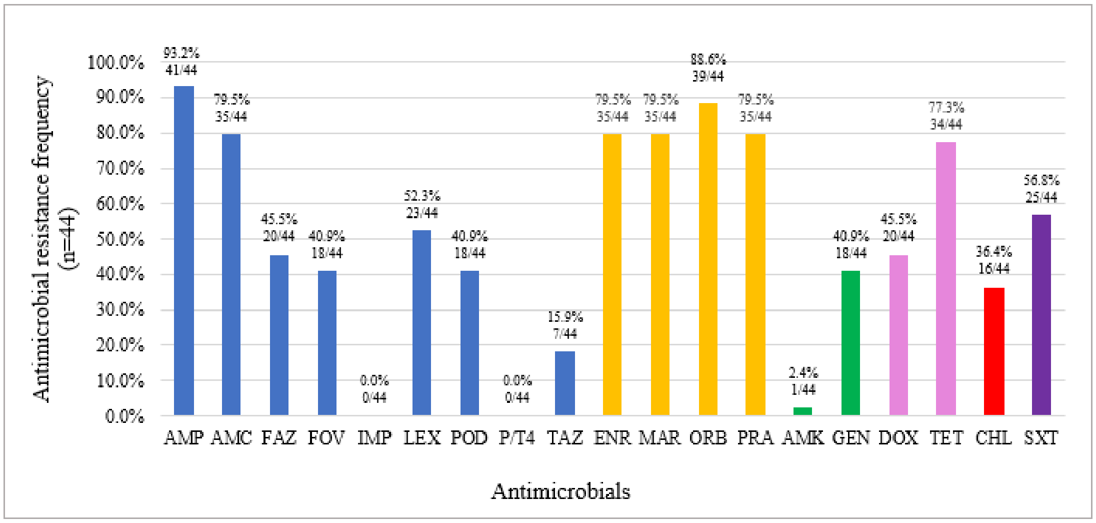

b

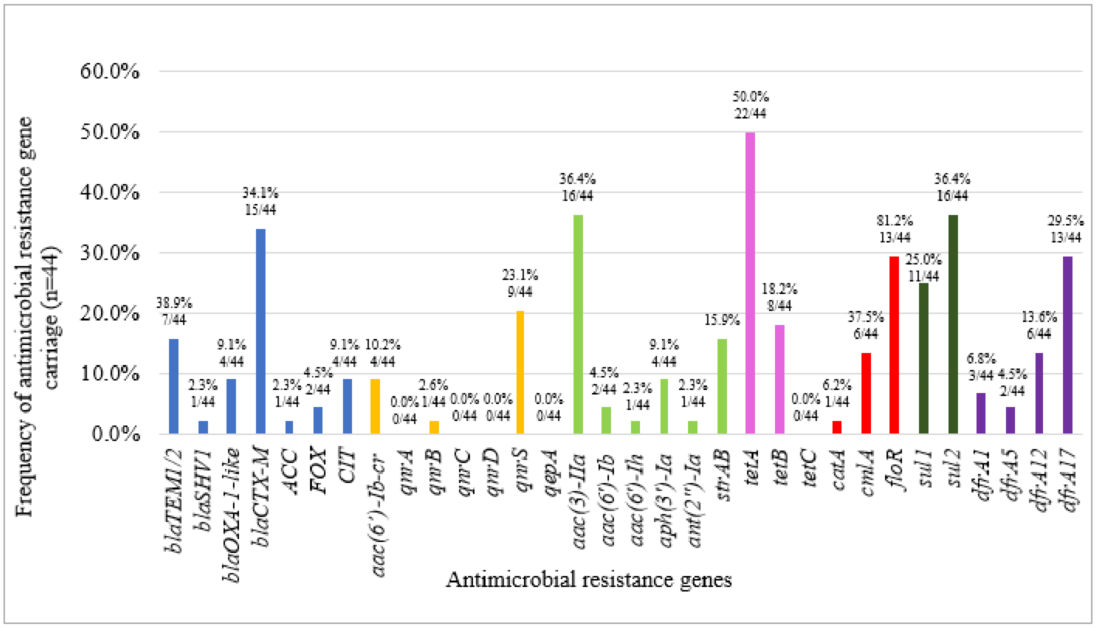

Supplementary Figure S1. Frequency and number of antimicrobial-resistant phenotypes and antimicrobial resistance genes. Figures present A) Frequency and number of antimicrobial-resistant phenotypes and B) Antimicrobial resistance genes detected in 44 third-generation cephalosporin and/or fluoroquinolone-resistant *Escherichia coli* isolated from pyometra, prostatic abscesses, and bloodstream infections in dogs and cats (AMP, ampicillin; AMC, amoxicillin/clavulanic acid; FAZ, cefazolin; FOV, cefovecin; IMP, imipenem; LEX, cephalexin; POD, cefpodoxime; P/T4, piperacillin/tazobactam; TAZ, ceftazidime; ENR, enrofloxacin; MAR, marbofloxacin; ORB, orbifloxacin; PRA, pradofloxacin; AMK, amikacin; GEN, gentamicin; DOX, doxycycline; TET, tetracycline; CHL, chloramphenicol; and SXT, sulfamethoxazole/trimethoprim)

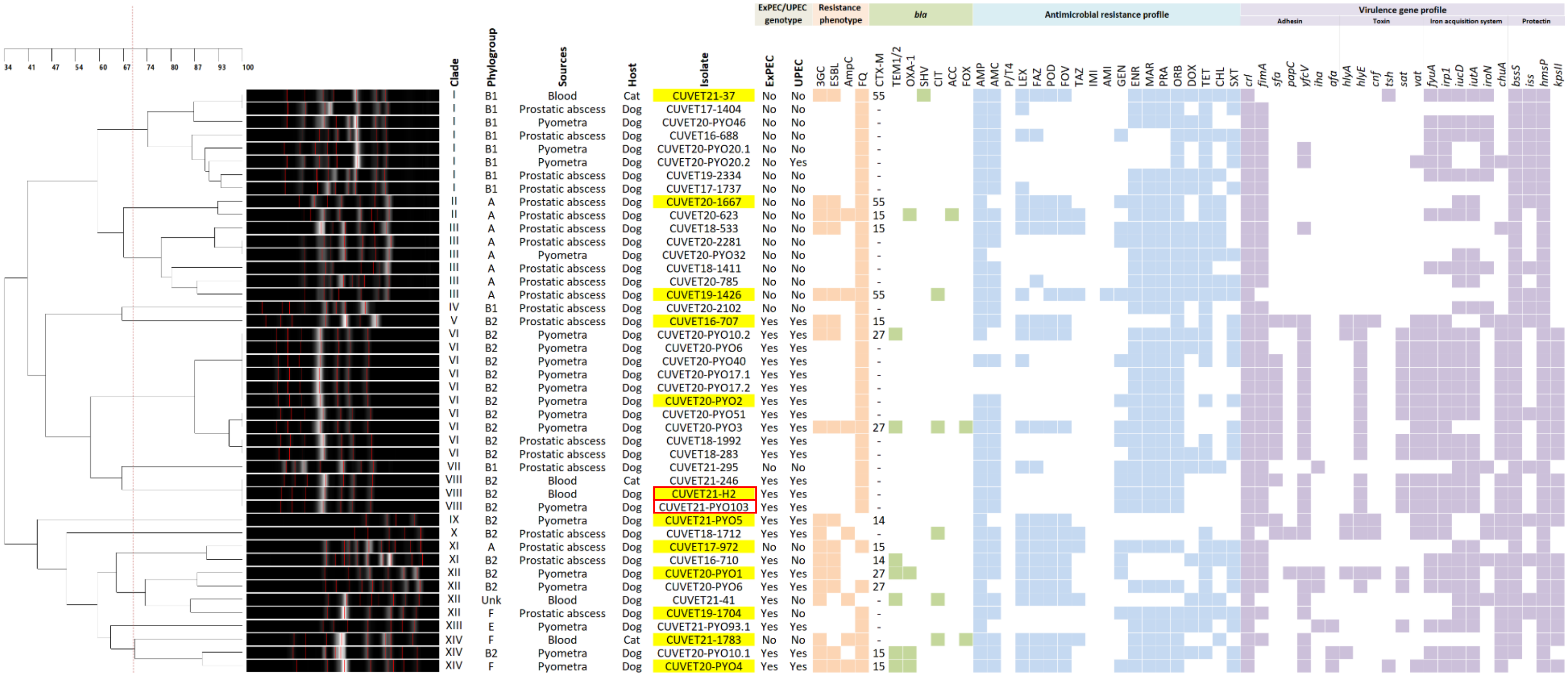

Supplementary Figure S2. Dendrogram based on repetitive extragenic palindromic sequence-PCR (REP-PCR) of 44 *Escherichia coli*. The dendrogram presents virulence genotypes, antimicrobial resistance profiles, and genetic features based on phylogroup and REP-PCR of 44 third-generation cephalosporin and/or fluoroquinolone-resistant *Escherichia coli* isolated from canine pyometra (n=19) and prostatic abscesses (n=20), and bloodstream infections in dogs and cats (n=5). Yellow highlight indicates representative strains subjected to whole-genome sequencing. Red borders indicate the two *E. coli* strains that were isolated from the same dog at the same time. (AMP, ampicillin; AMC, amoxicillin/clavulanic acid; FAZ, cefazolin; FOV, cefovecin; IMP, imipenem; LEX, cephalexin; POD, cefpodoxime; P/T4, piperacillin/tazobactam; TAZ, ceftazidime; AMK, amikacin; GEN, gentamicin; ENR, enrofloxacin; MAR, marbofloxacin; ORB, orbifloxacin; PRA, pradofloxacin; DOX, doxycycline; TET, tetracycline; CHL, chloramphenicol; SXT, sulfamethoxazole/trimethoprim)

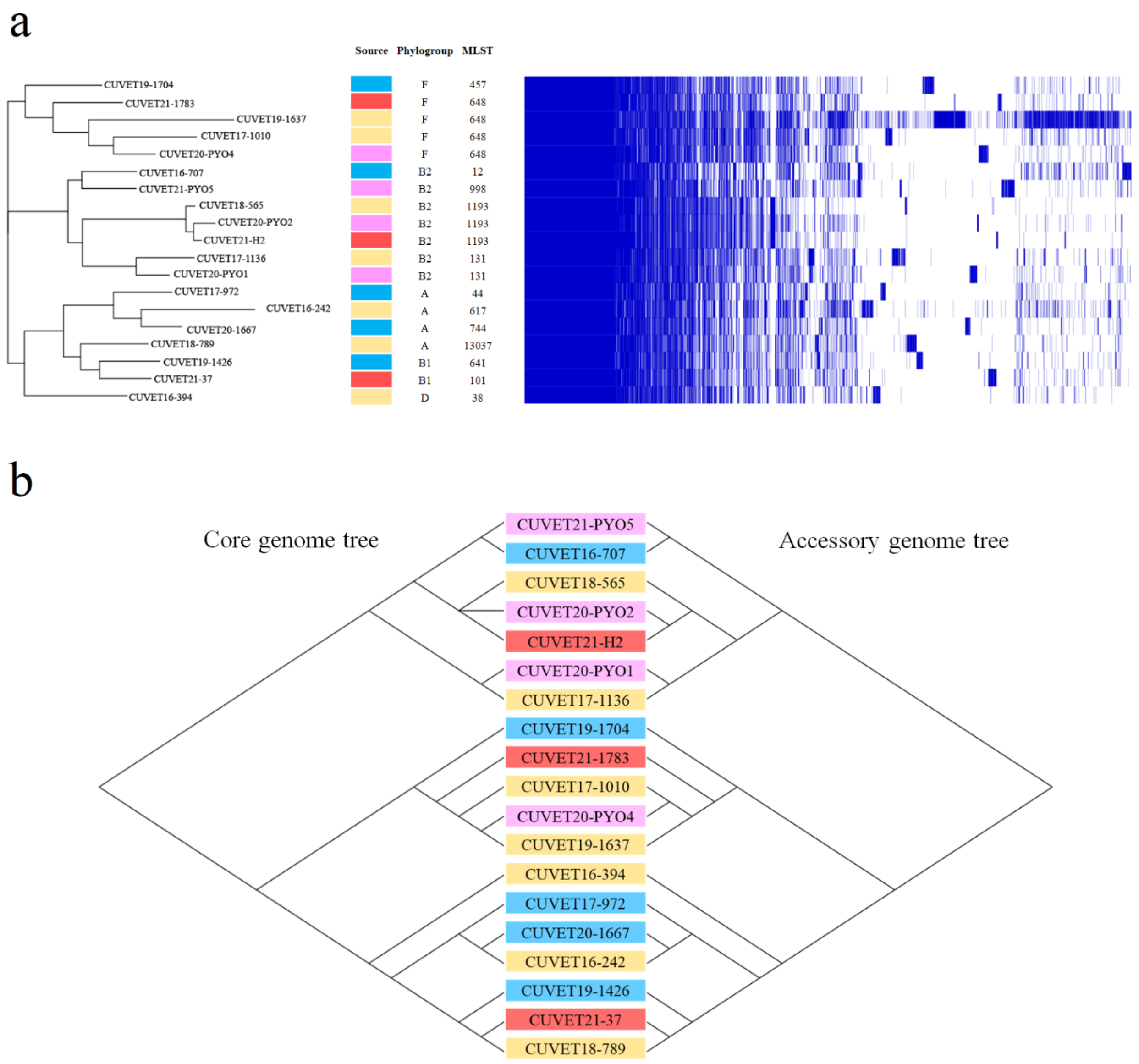

Supplementary Figure S3. Pan-genome-based binary tree and the relationship between coreSNP phylogeny and pan-genome analysis. A) A binary tree was constructed based on the presence or absence of accessory genes of 19 sequenced *Escherichia coli*. B) A comparison between coreSNP-based phylogeny and the accessory genome from the binary tree indicates a close relationship between chromosomal mutation and accessory gene acquisition. The pink band indicates *E. coli* isolated from pyometra, blue represents prostatic abscesses, red corresponds to bloodstream infections, and yellow denotes urinary tract infections.

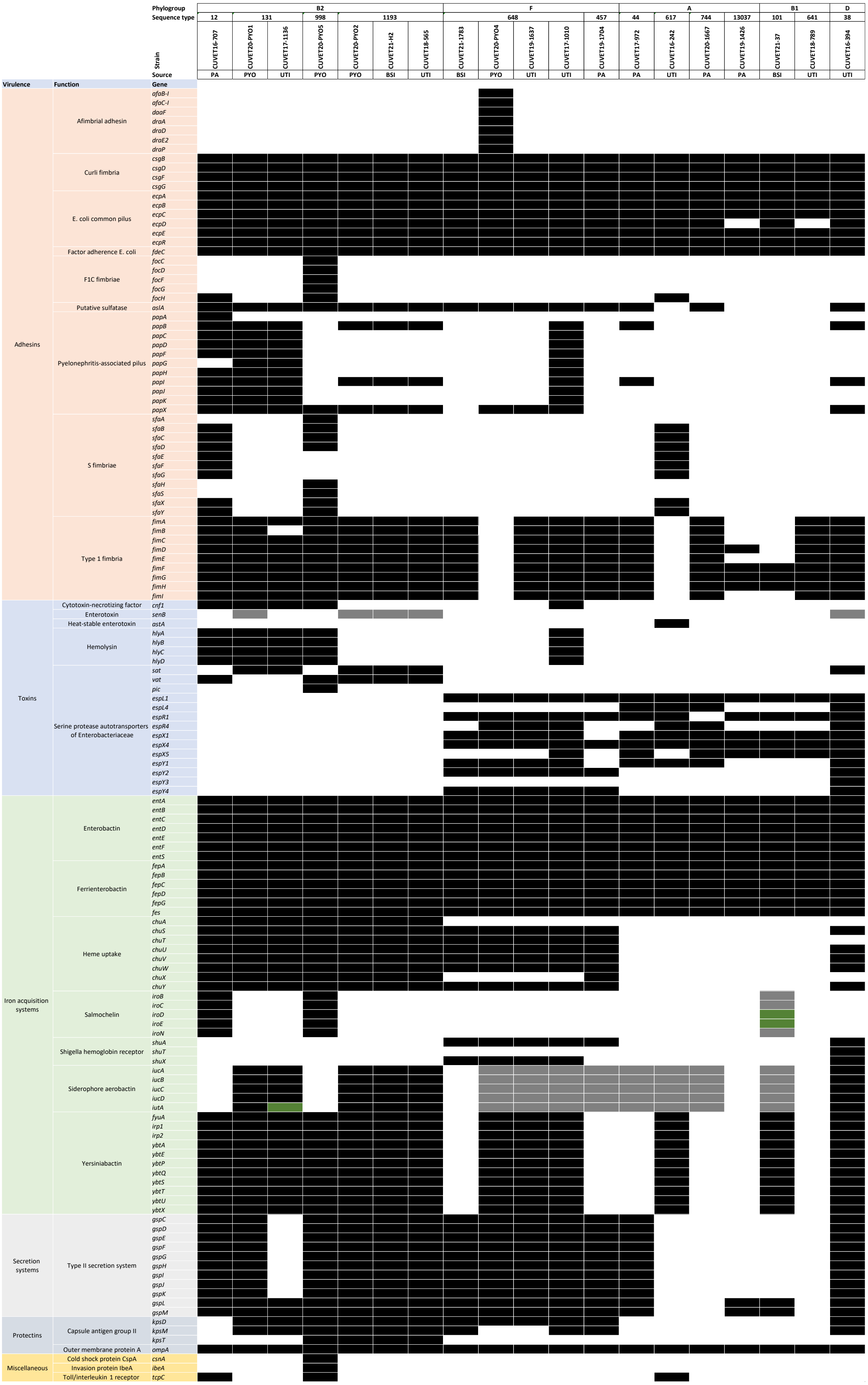
Phylogroup
B2
F
A
B1
D
Sequence type
12
131
998
1193
648
457
44
617
744
13037
101
641
38
Strain
CUVET16-707
CUVET20-PYO1
CUVET17-1136
CUVET20-PYO5
CUVET20-PYO2
CUVET21-H2
CUVET18-565
CUVET21-1783
CUVET20-PYO4
CUVET19-1637
CUVET17-1010
CUVET19-1704
CUVET17-972
CUVET16-242
CUVET20-1667
CUVET19-1426
CUVET21-37
CUVET18-789
CUVET16-394
Source
PA
PYO
UTI
PYO
PYO
BSI
UTI
BSI
PYO
UTI
UTI
PA
PA
UTI
PA
PA
BSI
UTI
UTI
Virulence
Function
Gene
Adhesins
Afimbrial adhesin
afaB-I
afaC-I
daaF
draA
draD
draE2
draP
Curli fimbria
csgB
csgD
csgF
csgG
E. coli common pilus
ecpA
ecpB
ecpC
ecpD
ecpE
ecpR
Factor adherence E. coli
fdeC
F1C fimbriae
focC
focD
focF
focG
focH
Putative sulfatase
aslA
Pyelonephritis-associated pilus
papA
papB
papC
papD
papF
papG
papH
papI
papJ
papK
papX
S fimbriae
sfaA
sfaB
sfaC
sfaD
sfaE
sfaF
sfaG
sfaH
sfaS
sfaX
sfaY
Type 1 fimbria
fimA
fimB
fimC
fimD
fimE
fimF
fimG
fimH
fimI
Toxins
Cytotoxin-necrotizing factor
cnf1
Enterotoxin
senB
Heat-stable enterotoxin
astA
Hemolysin
hlyA
hlyB
hlyC
hlyD
Serine protease autotransporters of Enterobacteriaceae
sat
vat
pic
espL1
espL4
espR1
espR4
espX1
espX4
espX5
espY1
espY2
espY3
espY4
Iron acquisition systems
Enterobactin
entA
entB
entC
entD
entE
entF
entS
Ferrienterobactin
fepA
fepB
fepC
fepD
fepG
fes
Heme uptake
chuA
chuS
chuT
chuU
chuV
chuW
chuX
chuY
Salmochelin
iroB
iroC
iroD
iroE
iroN
Shigella hemoglobin receptor
shuA
shuT
shuX
Siderophore aerobactin
iucA
iucB
iucC
iucD
iutA
Yersiniabactin
fyuA
irp1
irp2
ybtA
ybtE
ybtP
ybtQ
ybtS
ybtT
ybtU
ybtX
Secretion systems
Type II secretion system
gspC
gspD
gspE
gspF
gspG
gspH
gspI
gspJ
gspK
gspL
gspM
Protectins
Capsule antigen group II
kpsD
kpsM
kpsT
Outer membrane protein A
ompA
Miscellaneous
Cold shock protein CspA
csnA
Invasion protein IbeA
ibeA
Toll/interleukin 1 receptor
tcpC

Supplementary Figure S4. Virulence gene profiles of 19 sequenced *Escherichia coli* strains isolated from pyometra (PYO), prostatic abscesses (PA), bloodstream infections (BSI) and urinary tract infections (UTI) in dogs and cats, analyzed using the Virulence Factor Database (VFDB). Black, gray, and green indicate the location(s) of virulence genes on the chromosome, plasmids, and both chromosome and plasmids, respectively.

References

1. Ewers, C., Janßen, T., Kießling, S., Philipp, H. C. & Wieler, L. H. Molecular epidemiology of avian pathogenic *Escherichia coli* (APEC) isolated from colisepticemia in poultry. *Vet. Microbiol*. **104**(1-2), 91–101 (2004).
2. Maurer, J. J., Brown, T. P., Steffens, W. & Thayer, S. G. The occurrence of ambient temperature-regulated adhesins, curli, and the temperature-sensitive hemagglutinin tsh among avian *Escherichia coli*. *Avian. Dis.* **42**(1), 106–118 (1998).
3. Janßen, T. *et al.* Virulence-associated genes in avian pathogenic *Escherichia coli* (APEC) isolated from internal organs of poultry having died from colibacillosis. *Int. J. Med. Microbiol.* **291**(5), 371–378 (2001).
4. Vigil, P. D. *et al.* Presence of putative repeat-in-toxin gene *tosA* in *Escherichia coli* predicts successful colonization of the urinary tract. *mBio*. **2**(3), e00066-11 (2011).
5. Spurbeck, R. R. *et al*. *Escherichia coli* isolates that carry *vat*, *fyuA*, *chuA*, and *ycfV* efficiently colonize the urinary tract. *Infect. Immun*. **80**(12), 4115–4122 (2012).
6. Spurbeck, R. R. *et al*. Fimbrial profiles predict virulence of uropathogenic *Escherichia coli* strains: contribution of ygi and yad fimbriae. *Infect. Immun*. 79(12), 4753–4763 (2011).
7. Lopes, C. *et al*. Insights on the genetic features of endometrial pathogenic *Escherichia coli* strains from pyometra in companion animals: Improving the knowledge about pathogenesis. *Infect. Genet. Evol*. **85**, 104453 (2020).
8. Johnson, J. R. *et al*. Host characteristics and bacterial traits predict experimental virulence for *Escherichia coli* bloodstream isolates from patients with urosepsis. *Open. Forum. Infect*. **2**(3), ofv083 (2015).
9. Johnson, J. R. & O'Bryan, T. T. Detection of the *Escherichia coli* group 2 polysaccharide capsule synthesis gene *kpsM* by a rapid and specific PCR-based assay. *J. Clin. Microbiol*. **42**(4), 1773–1776 (2004).
10. Park, C. H., Robicsek, A., Jacoby, G. A., Sahm, D. & Hooper, D. C. Prevalence in the United States of *aac (6')-Ib-cr* encoding a ciprofloxacin-modifying enzyme. *Antimicrob. Agents. Chemother*. **50**(11), 3953–3955 (2006).
11. Tamang, M. D. *et al*. Emergence of multidrug-resistant *Salmonella enterica* serovar Typhi associated with a class 1 integron carrying the *dfrA7* gene cassette in Nepal. *Int. J. Antimicrob. Agents*. **30**(4), 330–335 (2007).
12. Keyes, K. *et al*. Detection of florfenicol resistance genes in *Escherichia coli* isolated from sick chickens. *Antimicrob. Agents. Chemother*. **44**(2), 421–424 (2000).
13. Karczmarczyk, M. *et al*. Characterization of antimicrobial resistance in *Salmonella enterica* food and animal isolates from Colombia: identification of a *qnrB19*-mediated quinolone resistance marker in two novel serovars. *FEMS. Microbiol. Lett*. **313**(1), 10–19 (2010).
14. Cattoir, V., Poirel, L., Rotimi, V., Soussy, C. J. & Nordmann, P. Multiplex PCR for detection of plasmid-mediated quinolone resistance *qnr* genes in ESBL-producing enterobacterial isolates. *J. Antimicrob. Chemother*. **60**(2), 394–397 (2007).
15. Kim, H. B. *et al*. Prevalence of plasmid-mediated quinolone resistance determinants over a 9-year period. *Antimicrob. Agents. Chemother*. **53**(2), 639–645 (2009).

16. Wang, M. *et al*. New plasmid-mediated quinolone resistance gene, *qnrC*, found in a clinical isolate of *Proteus mirabilis*. *Antimicrob. Agents. Chemother*. **53**(5), 1892–1897 (2009).
17. Cavaco, L. M., Hasman, H., Xia, S. & Aarestrup, F. M. *qnrD*, a novel gene conferring transferable quinolone resistance in *Salmonella enterica* serovar Kentucky and Bovismorbificans strains of human origin. *Antimicrob. Agents. Chemother*. **53**(2), 603–608 (2009).
18. Lanz, R., Kuhnert, P. & Boerlin, P. Antimicrobial resistance and resistance gene determinants in clinical *Escherichia coli* from different animal species in Switzerland. *Vet. Microbiol*. **91**(1), 73–84 (2003).
19. Guillaume, G., Verbrugge, D., Chasseur-Libotte, M. L., Moens, W. & Collard, J. M. PCR typing of tetracycline resistance determinants (Tet A–E) in *Salmonella enterica* serotype Hadar and in the microbial community of activated sludges from hospital and urban wastewater treatment facilities in Belgium. *FEMS. Microbiol. Ecol*. **32**(1), 77–85 (2000).
20. Karahutová, L., Mandelík, R. & Bujňáková, D. Antibiotic resistant and biofilm-associated *Escherichia coli* isolates from diarrheic and healthy dogs. *Microorganisms*. **9**(6), 1334 (2021).
21. Dallenne, C., Da Costa, A., Decré, D., Favier, C. & Arlet, G. Development of a set of multiplex PCR assays for the detection of genes encoding important beta-lactamases in *Enterobacteriaceae*. *J. Antimicrob. Chemother*. **65**(3), 490–495 (2010).
22. Grape, M., Motakefi, A., Pavuluri, S. & Kahlmeter, G. Standard and real-time multiplex PCR methods for detection of trimethoprim resistance *dfr* genes in large collections of bacteria. *Clin. Microbiol. Infect*. **13**(11), 1112–1118 (2007).
23. Akers, K. S. *et al*. Aminoglycoside resistance and susceptibility testing errors in *Acinetobacter baumannii-calcoaceticus* complex. *J. Clin. Microbiol*. **48**(4), 1132–1138 (2010).
24. Liu, J. H. *et al*. Detection and characterisation of CTX-M and CMY-2 β-lactamases among *Escherichia coli* isolates from farm animals in Guangdong Province of China. *Int. J. Antimicrob. Agents*. **29**(5), 576–581 (2007).
